# Interspecific and Intraspecific Hybrid Rootstocks to Improve Horticultural Traits and Soil-Borne Disease Resistance in Tomato

**DOI:** 10.3390/genes13081468

**Published:** 2022-08-17

**Authors:** Mean Vanlay, Song Samnang, Hee-Jong Jung, Phillip Choe, Kwon Kyoo Kang, Ill-Sup Nou

**Affiliations:** 1 Department of Horticulture, Suncheon National University, 255 Jungang-ro, Suncheon 57922, Jeonnam, Korea; 2 Department of Horticulture, PPS Co., Ltd., #51 Hagalro86beon-gil, Giheung-gu, Yongin-si 17096, Gyeonggi-do, Korea; 3 Division of Horticultural Biotechnology, Hankyong National University, Anseong 17579, Gyeonggi-do, Korea

**Keywords:** tomato rootstocks, *S. lycopersicum*, *S. habrochaites*, interspecific hybrids, intraspecific hybrids, horticultural trait, gene/loci, commercial rootstock

## Abstract

Tomato rootstocks are important to increase yield and control soil-borne pathogens, increasing vigor for a longer crop cycle and tolerance to biotic and abiotic stress. This study, conducted in the greenhouse of Sunchon National University during the period from 2019 to 2022, aimed to identify local soil-borne-disease resistant interspecific and intraspecific tomato hybrid rootstocks. The 71 interspecific hybrids (*S. lycopersicum* × *S. habrochaites*) showed that the germination vigor (GV) was less than Maxifort, except for several combinations. The germination rate (GP) of cross-species hybrids showed a different pattern according to the hybrid combinations, of which three combinations showed less than 30%. The horticultural traits, such as GV and GP, of the intraspecies hybrid (*S. l* × *S. l*) combination were significantly improved compared to that of Maxifort. In 71 combinations (*S. l* × *S. h*) and 25 combinations (*S. l* × *S. l*), MAS was used to evaluate the resistance of eight genes related to soil-borne pathogens, four genes related to vector-mediated pathogens, and three genes related to air-borne pathogens. The results showed that the new hybrid combination had improved resistance over the commercial-stock Maxifort. Therefore, interspecies and intraspecies hybrid techniques for breeding commercial rootstocks can be utilized as a way to improve horticultural properties and resistance to soil-borne diseases in tomato.

## 1. Introduction

Tomatoes (*Solanum lycopersicum,* Solanaceae; 2n = 2x = 24, 25, 26) are an important vegetable crop grown worldwide from temperate to tropical and subtropical regions and are particularly valued for their nutritional qualities [1,2,3]. World production of fresh tomatoes for 2020 was about 182 million tons, planted on 4.76 million hectares in 168 countries [4]. Tomatoes are supposed to have originated in western South America and were domesticated in Central America. The tomato plant has a number of distinguishing properties, including fleshy fruit, a sympodial stalk, and compound leaves, which are not seen in other model plants (such as rice and *Arabidopsis*) [5]. The wild tomato *Solanum habrochaites* S. Knapp et D.M. Spooner (formerly *Lycopersicon hirsutum* Dunal) is the most resilient and has the showiest floral displays of all the wild tomato species. This species can be found on the western slopes of the Andes at heights ranging from 400 to 4000 m, from central Ecuador to central Peru [6]. Peralta et al. [7] found that the cultivated tomato is closely related to 13 wild *Solanum* species, all of which can be crossed with tomatoes with varying degrees of difficulty. All wild tomatoes are diploid (2n = 24), may be crossed with cultivated tomatoes and serve as a breeding source for desirable qualities such as enhanced production yield, fruit quality, disease, and abiotic stress resistance. Wild tomato species are very useful in evolutionary research [8,9]. The current effort on the tomato-genome-sequencing project has yielded significant data for tomato research [5]. This wild species could be a source of unique tomato-breeding genes [10]. Implementing techniques to maintain wild germplasm is necessary for making the best use of genetic resources in current and future breeding. Plant breeding aims to increase the probability of developing and identifying superior genotypes that will result in successful new cultivars. “In other words, they will have all of the desirable characteristics/traits that are required for usage in a manufacturing system” [11]. The breeding process necessitates: (i) the identification of variable germplasm; (ii) hybridization to combine genetic materials from various sources into a single entity; (iii) the selection of superior genotypes with a favorable combination of characteristics; and (iv) the multiplication of stable cultivars prior to the commercial release of a new cultivar. The cultivated *Solanaceae* parent is usually used as the female and the wild species is the pollen donor in breeding programs, and the cross’s success is determined by the percentage of fruit set, the number of seeds per fruit, and the percentage of the germination of the F_1_ seed [12,13,14]. In the *Solanaceae*, using a hybrid with at least one parent that preserves a high complement of wild-species DNA is common, and the most extensively used commercial hybrid rootstock for tomatoes are *Solanum lycopersicum* L. (tomato) and *Solanum habrochaites* S. Knapp and D.M. Spooner (wild species). The tomato was one of the first crops for which molecular markers were proposed as an indirect breeding-selection criterion [15,16,17]. Using molecular markers, the success of creating interspecific hybrids may be simply determined [18]. Crosses between cultivated tomatoes and their wild relatives result in plants with higher growth, which is regarded as vigor, and combines numerous features (e.g., disease or pest resistance, salt tolerance, cold tolerance) [19,20,21,22,23]. In recent years, a slew of new tomato rootstocks has hit the market. However, only a handful are frequently employed in practice because of their superior performance or the market availability of seeds [24,25]. Seedling emergence, uniformity of growth, and stem diameter are all affected by the quality of rootstock seed [26,27]. More than 200 diseases caused by a pathogenic fungus, bacteria, viruses, or nematodes can affect tomatoes [28]. Plant diseases are responsible for up to 26% of yield loss in worldwide agriculture, and crop failure can occur at any moment [29]. Genetic factors, maternal environment, and fruit harvest time all have an impact on seed yield and quality [30]. The consistent germination of wild species and hybrids (cultivated tomato x wild) was tested, and there was a lot of diversity [31]. Wild species and hybrids (cultivated tomato x wild) have a wide range of germination rates, ranging from 8% to 86% [31]. According to Tikoo et al. [32], *S. l* or interspecific hybrids (*S. l* × *S. h*) are commonly utilized as tomato rootstock in *Solanaceous* vegetables. The specific objectives of this study were: (1) To develop *S. l* and *S. h* lines with a high germination rate, excellent germination energy, high seed harvesting, strong plant vigor and deep roots. (2) To develop lines with multiple resistance (e.g., *Verticillium* wilt, *Fusarium* wilt, *Bacteria* wilt, and *Fusarium* crown and root rot). (3) To develop new varieties of tomato rootstocks that have multiple resistant to soil-borne disease, high germination, strong vigor and biotic and abiotic stress tolerance.

## 2. Materials and Methods

This research was conducted in the greenhouse at Sunchon farm practice of Sunchon National University, South Korea. The experiment was carried out for 3 years from 2019 to 2022.

### 2.1. Plant Materials

#### 2.1.1. Tomato Selfing

A total of 43 different tomato cultivars were used for this study: JTS01 to JTS43 imported from Japan (Taki seed company Tokyo, Japan), JTS14, JTS15, JTS41, JTS42 and JTS43, collected from South Korea (Nongwoo Bio company, Anseong, Korea), and the wild tomato species seeds obtained were 44 different tomato accessions self-compatible from the United States Department of Agriculture (USDA) germplasm. All seeds were sown in 50-cell (cell volume of 39 mm × 45 mm) trays containing cocopit soil mix in the greenhouse and all plants were grown in the greenhouse at Sunchon farm practice. The pollen falls within the flower to pollinate itself by natural (wind) and using a handle vibrator.

#### 2.1.2. Tomato Crossing and Production of F1 Hybrids

Specific crosses were conducted using 17 inbred parental lines as female parents (*S. l*), 5 accessions of wild species as male parents (*S. h*), and 2 inbred lines as male parents (*S. l*) The selection of these parents was based on growth traits such as high germination, vigor, many fruit sets, high seed products and diseases resistance, especially soil-borne diseases, and tolerance to abiotic stresses. All crosses were performed by hand pollination. The female plants (*S. l*) were emasculated before the flower opened (removing stamens, petals and sepals), typically a day before the anthesis (evening time). Pollen was collected from the male parent (*S. h*). A handle vibrator was used to collect pollen on the tip (volume 1.5 mL) and apply pollen to the stigma surface (morning time). All the crosses were made in the morning between 09:00 to 11:30 a.m. local time. After pollination, all flowers were tagged with labels that included names and dates. Harvesting of tomato fruit was carried out daily until the end of the season. The fruit set rate was determined as the total number of fruits divided by the total number of pollinated flowers on each plant. Seed yield was determined as the total seed obtained divided by the total fruit harvested from each plant. The F_1_ hybrids were transplanted on both sides of the bed (width: 1.2 m; row space: 0.8 m). The in-row distance between plants was 30 cm. Each experimental unit (EU) consisted of 2 plants. All cultural practices (fertilization, irrigation, weeding, and disease and insect control) were performed as recommended for commercial greenhouse tomato production.

#### 2.1.3. Evaluated Traits and Marker-Assisted Selection (MAS)

The germination percentage (GP) was counted at the time germination was completed (100 seeds per line were sown in then-rolled towel papers) [33]. The germination vigor was measured by counting the number of seedlings emerging daily (7–14 days) from the day of planting the seeds in a medium till the time germination was complete (one hundred seeds were sown in 105-cell trays containing cocopit soil mix). Germination Index (GI) or Germination Vigor (GV) was computed by using the following formula: GV = n/d (n: number of seedlings emerging on the day, d: the day after sowing) [33]. The GV rating was scored for each line/hybrid combination based on a 1 to 9 scale (note, 1 = very weak, 2 = very weak to weak, 3 = weak, 4 = weak to medium, 5 = medium, 6 = medium to strong, 7 = strong, 8 = strong to very strong, 9 = very strong) following Juss and Shaw et al. [34]. Plant growth measurements, internode length (IL) F_1_ (from the base to the leaf 3rd, 5th, 7th, 9th, 11th), total root length (TRL) (from the root collar to the end of the root by meters), root fresh mass (RFM) (after washing for 3 h with scales), were measured 60 days after transplanting (DAT). The seedling length (SL) and the plant height (PH) (from the base to the end of the stem in meters), seedling stem diameter (SSD), and plant stem diameter (PSD) (from cotyledon to 1st leaf, the leaf 9th to 10th internode from the base by digimatic caliper) were measured twice, 30 days after sowing (DAS) and 60 days after transplanting (DAT). The method of root collection was to dig from the ground by spraying water gradually because all tomato plants were planted on the ground directly. The yield was measured by the average number of seeds per fruit (ANSF) for each plant (harvesting of tomato fruit was carried out daily until the end of the season for *S. h*, but *S. l* was collected only 2 or 3 times for good fruit (big size, no blossom-end rot, no cracked fruits and disease). The methods of marker-assisted selection (MAS) were performed based on HRM curve method and judged with resistance or susceptibility instead of trait values [35]. Then, the experiment evaluated resistance with a marker such as: *Fusarium wilt; I2* [36], *Fusarium wilt*; *I3* [37], *Verticillium wilt; Ve2* [38], *Fusarium crown and root rot*; *J3* [39], *Corky root rot*; *py1* [40], *Root-Knot nematode*; *Mi23* [41], *Bacterial wilt; Bw6* and *Bw12* [42], *Tomato Spotted wilt virus*; *TSWV* or *Sw5* [43], *Tomato mosaic virus*; *ToMV* or *Tm2a* (Unpublicized data), *Tomato yellow leaf curl virus*; *TYLCV* or *Ty1* [44], *Tomato yellow leaf curl virus*; *TYLCV* or *Ty2* [45], *Late blight*; *Ph3* [46], *Gray leaf spot*; *Sm-565* [47] and *Leaf mold*; *Cf9* [48].

### 2.2. DNA Extraction

For extraction of genomic DNA, young leaves (1 g) of 172 tomato cultivars, wild species, and F1 hybrids were collected and genomic DNA was isolated by CTAB method [49]. PCR was performed in a total volume of 10 µL containing 2 µL of genomic DNA, 0.5 µL of forward and 0.5 µL of reverse primers (10 pmol), 5 µL of Prime Taq Premix and 2 µL of distilled water. The reaction condition was as follows: samples were primarily denatured at 95 °C for 5 min; followed by 30 cycles of 95 °C for 30 s, 60 °C for 30 s, and 72 °C for 30 s and final elongation at 72 °C for 5 min in a GenAmp PCR system 9700 (Applied Biosystems, Seoul, Korea). The amplicon was run on a 1.2% agarose gel. PCR conditions for *Ty2* and *cf9* were: initial denaturation at 95 °C for 5 min followed by denaturation at 95 °C for 30 s, annealing at 60 °C for 30 s, elongation at 72 °C for 30 min repeated for up to 30 cycles and final elongation at 72 °C for 5 min. Finally, the reaction mixture was cooled down to 4 °C and the amplicon was loaded on 1.2% agarose gel concentration.

### 2.3. HRM Analysis

The PCR reactions were carried out in a total volume 10 µL containing 1.5 µL of genomic DNA, 1 µL of each primer, 5 µL of HS Prime LP Premix (GENETBIO, Daejeon, Korea), 0.1 µL of forward and 0.5 µL of reverse primers (10 pmol), 0.5 µL probe (10 pmol), 0.3 µL SYTO 9 fluorescent dye and 2.6 µL of distilled water. The HRM condition for *I2* and *I3*, *Ve2*, *J3*, *py1*, *Mi23*, *Bw6* and *Bw12*, *Sw5*, *Tm2a*, *Ty1*, *Ph3* and *sm-565* was: an initial preincubation at 95 °C for 5 min followed by 40 cycles of 95 °C for 10 s, annealing at 64 °C and 56 °C (−1 °C) for 15 s under touchdown command, and 72 °C for 15 s. HRM data were recorded by four readings per 1 °C at the final step after 60 s at 95 °C, 60 s at 40 °C, and 1 s at 97 °C. HRM curve analysis was conducted using LightCycler 96 software (Roche, Mannheim, Germany) at 75% discrimination for both delta Tm and curve shape with a 0.2 positive/negative threshold level.

## 3. Results

### 3.1. Horticultural Traits and Marker Selection of S. lycopersicum

**Figure 1:**
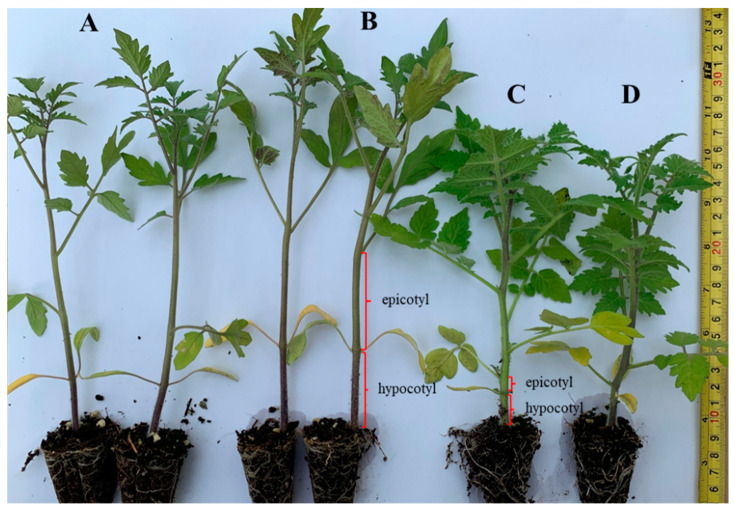
The hypocotyl length and epicotyl length of *S. l* and *S. h* at 30 DAS. A: JTS01-3, B: JTS21-1, C: SN-14, D: SN-41.

**Figure 2:**
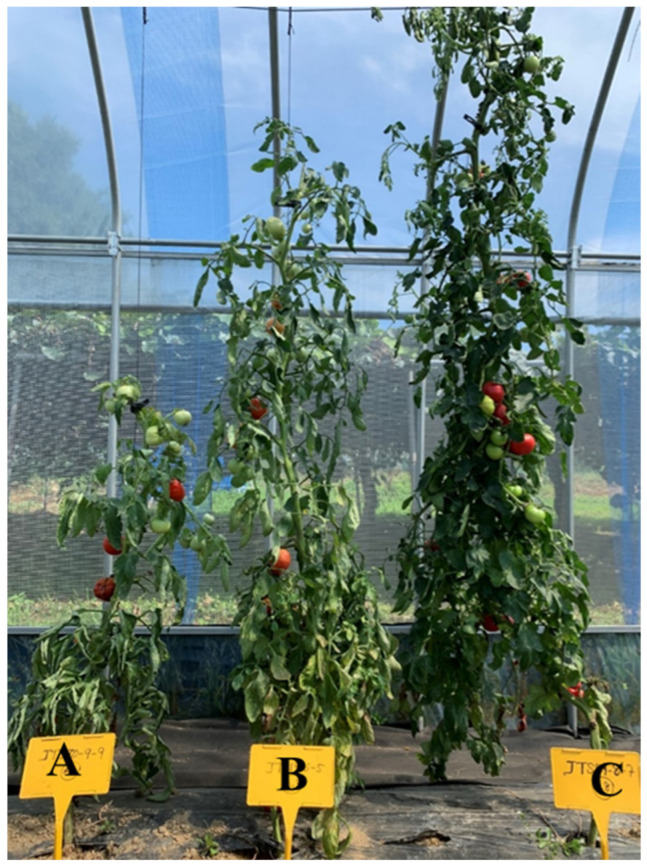
The plant height of *S. l* at 60 DAT. A: JTS29-3, B JTS18-2, C: JTS20-2. Plant type of *S. l.* (**A**): determinate (D), (**B**,**C**): indeterminate (ID).

A total of 101 lines were developed from 43 cultivars. The selection was based on growth traits such as germination percentage (GP), germination vigor (GV), a high number of fruit sets, high seed products, and disease resistance, especially to soil-borne diseases. In the experiments, the hypocotyl length and epicotyl length of *S. l* were longer than *S. h* (Figure 1). Most of the study lines had indeterminate (ID) growth types in 70 lines (69.30%), with only 31 lines (30.70%) being determinate (D) (Table 1, Figure 2). The average number of seeds per fruit (ANSF) was detailed; these were more than 19.5 seeds/fruit on 64 lines (63.37%), and less than 19.5 seeds/fruit on 37 lines (36.63%) (Table 1). The maximum ANSF was recorded by JTS32-2 (75 seeds/fruit) and the minimum ANSF was recorded by JTS15-1 (2 seeds/fruit) (Table 1). The final germination percentage (GP) of the seeds, which were germinated without any pretreatment (control), ranged considerably (from 31% to 99%), depending on the cultivar. The GP was less than 50% for 6 lines (5.94%), between 50% to 85% for 39 lines (38.61%), and more than 85% for 56 lines (55.45%) (Table 1). The following germination vigor (GV) values were found: 1 = very weak were 6 lines (5.94%), 3 = weak were 10 lines (9.90%), 5 = medium were 27 lines (26.74%), 7 = strong were 29 lines (28.71%), and 9 = very strong were 29 lines (28.71%), as shown in Table 1. The seedling length (SL) and the seedling stem diameter (SSD) were measured at 30 DAS. The SL was described as: longer than 17.50 cm were 61 lines (61.40%), and shorter than 17.50 cm was 40 lines (39.60%) (Table 1). The maximum of SL was recorded by JTS21-1 (26 cm) and the minimum of SL was recorded by JTS06-4 (12 cm) (Table 1). The seedling stem diameter (SSD) was as follows: bigger than 3.99 mm were 61 lines (61.40%), and smaller than 3.99 mm were 40 lines (39.60%) at 30 DAS (Table 1). There were three types of plant stem diameter (PSD) and plant height (PH): small/short, medium, big/high at 60 DAT. The PSD was described as: smaller than 15 mm were 30 lines (29.70%), between 15 mm to 18 mm were 49 lines (48.51%), and bigger than 18 mm were 22 lines (21.79%) (Table 1). The PH was as follows: shorter than 150 cm were 37 lines (36.63%), between 150 to 180 cm were 33 lines (32.67%), and bigger than 180 cm were 31 lines (30.70%) (Table 1, Figure 2).

Marker-assisted selection (MAS) was used to identify quality traits and disease resistance. All generations were selected as homozygote/heterozygote based on DNA markers, especially soil-borne pathogens (Table 1).

### 3.2. Horticultural Traits and Marker Selection of Solanum habrochaites

A total of 42 lines were selected from 44 accessions. The selection was based on growth traits such as germination percentage (GP), germination vigor (GV), many fruit sets, and high seed products. The seedlings of *S. h* had purple hypocotyls above the soil level, and the length of the hypocotyl was shorter than S. l under the same condition (Figure 1). In the experiments, the *S. h* were indeterminate (ID). In the experiments, *S. h* had genes resistant to *Fusarium* crown and root rot (*J3*), tomato spotted wilt virus (*Sw5/TSWV*), tomato yellow leaf curl virus (*Ty2/TYLCV*), late blight (Ph3), gray leaf spot (*Sm-565*), and *Fusarium* wilt (I2); root-knot nematode (Mi23) and leaf mold (Cf9) were not amplified (Table 2). The germination percentage (GP) of *S. h* was as follows: less than 50% was recorded by SN-15 (2.38%), between 50 to 85% were 14 lines (33.33%), and more than 85% were 27 lines (64.29%), as shown in (Table 2). The germination vigor (GV) of *S. h* was described; 3 = weak were eight lines (19.05%), 5 = medium were 13 lines (30.95%), 7 = strong were 13 lines (30.95%), and 9 = very strong were eight lines (19.05%) (Table 2). The seedling length (SL) of *S. h* was measured from the base to the end of the stem at 30 DAS. The SL of *S. h* was as follows: longer than 10.5 cm were 21 lines (50%), and shorter than 10.5 cm were 21 lines (50%) (Table 2). The maximum SL was recorded by SN-31 (16 cm), and the minimum SL was recorded by SN-10 (7 cm) (Table 2). The seedling stem diameter (SSD) was measured between cotyledon to 1st leaf at 30 DAS. The SSD of *S. h* was as follows: bigger than 3.01 mm were 22 lines (52.38%), and smaller than 3.01 mm were 20 lines (47.62%) (Table 2). The maximum SSD was recorded by SN-31 (3.66 mm), and the minimum SL was recorded by SN-11 (2.23 mm) (Table 2). In the experiments, the plant height (PH) and the plant stem diameter (PSD) of *S. h* were non-significantly different, except SN-14 (14.85 mm) at 60 DAT (Table 2). In this research, high fruit setting and many seeds per fruit were target-specific for commercial rootstock. The average number of seeds per fruit (ANSF) of *S. h* was: less than 14.5 seeds/fruit for 27 lines (64.29%), and more than 14.5 seeds/fruit for 15 lines (35.71%) (Table 2). ANSF values were highest in SN-12 lines (33 seeds/fruit) and lowest in SN-14 and SN-37 lines (6 seeds/fruit) (Table 2).

### 3.3. Genetic Control and Horticultural Traits of F1 hybrids

**Figure 3:**
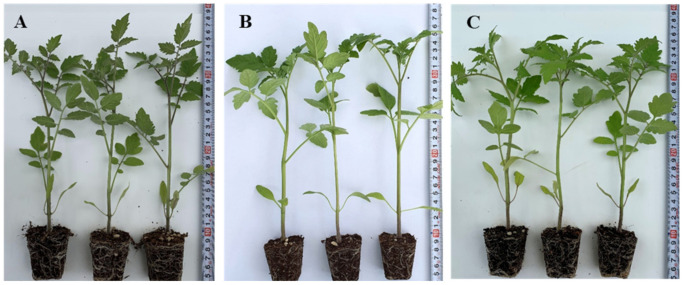
The morphology of tomato seedlings at 30 DAS, (**A**): interspecific hybrid (64 = JTS21-3 × SN-33), (**B**): intraspecific hybrid (78 = JTS35-3 × JTS33-3), (**C**): commercial rootstock (Maxifort).

**Figure 4:**
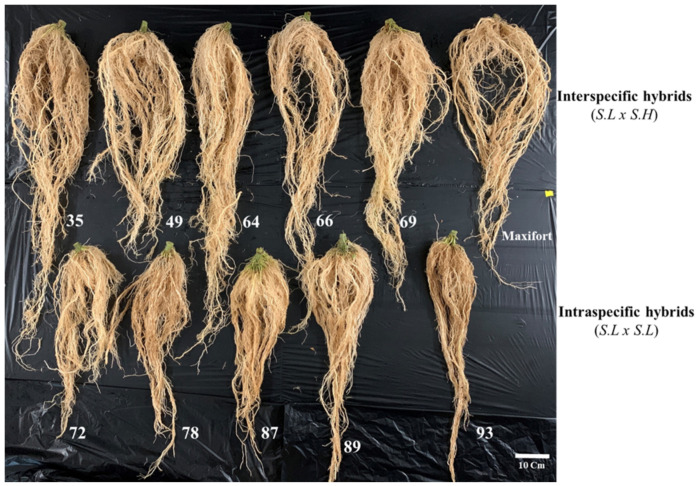
The morphology of tomato interspecific-hybrids (*S. l* × *S. h*) and intraspecific-hybrids (*S. l* × *S. l*) hybrid rootstocks at 60 days after transplanting, 35 = (JTS21-3 × SN-08), 49 = (JTS21-3 × SN-20), 64 = (JTS21-3 × SN-33), 66 = (JTS25-5 × SN-33), 69 = (JTS28-4 × SN-33), 72 = (JTS01-3 × JTS33-3), 78 = (JTS35-3 × JTS33-3), 87 = (JTS11-4 × JTS35-4), 89 = (JTS35-3 × JTS35-4), 93 = (JTS25-5 × JTS35-4), Maxifort (Control).

A total of 96 new hybrid seed products, 71 interspecific hybrids (*S. l* × *S. h*) and 25 intraspecific hybrids (*S. l* × *S. l*) were identified (Table 3, Table 4). During crossing time, the fruit setting and seed yield of interspecific hybrids were determined by the phenotype of female parents. As a result, JTS01-3 produced high fruit setting and high seed product even though it had a small fruit size. In contrast, JTS37-3 had a larger fruit size but less fruit setting, and seed products. In the experiments, we observed that the female parent of D type was better than the female parent of ID-type in hybrid rootstock with respect to horticultural traits, such as germination percentage, germination vigor, and stem girth (Table 4). In experiments, the germination speed and seedling vigor of intraspecific hybrids were better than interspecific hybrids, respectively. The GP was also significantly affected by female-and-male-parent interaction, revealing genetic variation among hybrids for germination response. As a result, the GP of commercial rootstock (Maxifor) was only 85% (Table 4). The GP of intraspecific hybrids was detailed: there were 98% three new hybrid combinations and 100% 23 new hybrid combinations (Table 4). The GP of interspecific hybrids was: more than 85% were 38 new hybrid combinations (37.62%), and lower than 85% were 63 new hybrid combinations (62.38%) (Table 4). In experiments, GV was important for commercial breeding such as rootstock grafting. The evaluation of GV was based on a 1-9 scale. The GV of intraspecific hybrids was described: 7 = strong were five new hybrid combinations (20%), and 9 = very strong, were 20 new hybrid combinations (80%) (Table 4). The GV of F_1_ in interspecific hybrids was described as follows: 1 = very weak were seven new hybrid combinations (9.86%), 3 = weak were 19 new hybrid combinations (26.76%), 5 = medium were 38 new hybrid combinations (53.52%) and 7 = strong were seven new hybrid combinations (9.86%). Therefore, the Maxifort was at a 7 on the scale and most of the intraspecific hybrids were at a 9 (Table 4, Figure 3). The SSD was measured between cotyledon to 1st leaf at 30 DAS. The SSD of Maxifort was 4.25 mm (Table 4). The SSD of interspecific hybrids was as follows: smaller than (3.99 mm) were three new hybrid combinations (12%), bigger than (4.00 mm) were 22 new hybrid combinations (88%) (Table 4). The SSD of interspecific hybrids was as follows: smaller than (3.99 mm) were 53 new hybrid combinations (74.65%), and bigger than (4.00 mm) were 18 new hybrid combinations (25.35%) (Table 4). The seedling length (SL) was measured from the base to the end of the stem at 30 DAS. The SL of Maxifort was 23 cm (Table 4). The SL of intraspecific hybrids was as follows: shorter than 18.5 cm were 7 new hybrid combinations (28%), and longer than 18.5 cm were 18 new hybrid combinations (72%) (Table 4). The SL of interspecific hybrids was: shorter than 18.5 cm were 46 new hybrid combinations (64.79%), longer than 18.5 cm were 25 new hybrid combinations (35.21%) (Table 4). The PH of Maxifort was 257 cm (Table 4). All the PH of interspecific hybrids were higher than intraspecific hybrids. The PH of intraspecific hybrids was as follows: lower than 199 cm were 20 F_1_ new combinations (80%), and higher than 199 cm were five new hybrid combinations (20%) (Table 3). The PH of interspecific hybrids was: lower than 249 cm were 13 new hybrid combinations (18.31%), higher than 249 cm were 58 new hybrid combinations (81.69%) (Table 4). The plant stem diameter (PSD) of new hybrid combinations and Maxifort were measured twice (between cotyledon to 1st leaf and the 9th to 10th leaf at 60 DAT). The PSD between cotyledon to 1st leaf of intraspecific hybrids was smaller than 14.30 mm. Interspecific hybrids were as follows: smaller than 15 mm were 43 new hybrid combinations (60.56%), bigger than 15 mm were 28 new hybrid combinations (39.44%), and Maxifort was 15.20 mm (Table 4). The PSD between the 9th and 10th leaf of intraspecific hybrids was described as follows: smaller than 15 mm were eight new hybrid combinations (32%), between 15 to 18 mm were 18 new hybrid combinations (56%), bigger than 18 mm 3 new hybrid combinations (12%); intraspecific hybrids were as follows: smaller than 15 mm was recorded by JTS07-2 × SN-08 (1.41%), between 15 to 18 mm were 55 new hybrid combinations (77.46%), bigger than 18mm were 15 new hybrid combinations (21.13%), and Maxifort was 20.16 mm, as shown in Table 4. Internode length is an important agronomic characteristic affecting plant architecture and crop yield. The IL was measured from the base to the 3rd leaf, 5th leaf, 7th leaf, 9th leaf, and 11th leaf at 60 DAT. In the study, we selected new hybrid combinations that had a short internode length; therefore, as a result, among 71 of the interspecific hybrids, there were 19 new hybrid combinations (JTS01-3 × SN-42), (JTS05-2 × SN-42), (JTS11-4 × SN-42), (JTS28-4 × SN-42), (JTS09-4 × SN-08), (JTS21-3 × SN-08), (JTS21-3 × SN-08), (JTS28-4 × SN-08), (JTS05-2 × SN-20), (JTS16-3 × SN-20), (JTS25-4 × SN-20), (JTS27-2 × SN-20), (JTS28-4 × SN-20), (JTS01-3 × SN-33), (JTS21-3 × SN-33), (JTS25-4 × SN-33), (JTS27-2 × SN-33), (JTS28-4 × SN-33), and (JTS33-3 × SN-33). These internode lengths of 19 new hybrid combinations were similar to Maxifort and other new hybrid combinations of interspecific hybrids were longer than Maxifort (Table 4). The IL of three new intraspecific hybrid combinations (JTS01-3 × JTS33-3, JTS05-2 × JTS33-3, JTS37-3 × JTS35-4) were similar to Maxifort and other new hybrid combinations of intraspecific hybrids were longer than Maxifort (Table 4). The root system of interspecific hybrids was more than intraspecific hybrids (Figure 4). The total root length (TRL) of Maxifort was 75 cm, and the root fresh mass (RFM) was 258.56 g at 60 DAT (Table 4). As a result, all the TRL of intraspecific hybrids were shorter than Maxifort, and the RFM of intraspecific hybrids was lighter than Maxifort, except (JTS35-3 × JTS35-4) was heavier than Maxifort (Table 4). All the RFM of interspecific hybrids were heavier than Maxifort. The TRL of interspecific hybrids was described as follows: 11 new hybrid combinations (15.50%) were shorter than Maxifort and 60 new hybrid combinations (84.50%) were heavier than Maxifort (Table 4).

Hybrid tomato varieties have multiple disease resistances, especially to soil-borne pathogens, such as Maxifort being resistant to *Fusarium wilt* (*I2*), *Verticillium wilt* (*Ve2*), *Fusarium crown and root rot* (*J3*), Root-Knot nematode (*Mi23*), *Tomato Spotted wilt virus* (*Sw5/TSWV*), *Tomato mosaic virus* (*Tm2a/ToMV*), and *Leaf mold* (*Cf9*). As a result, all new hybrid combinations were more resistant than commercial rootstock Maxifort, except only one new hybrid combination (JTS35-3 × SN-08) had the same resistance as Maxifort but to different diseases (Table 3).

## 4. Discussion

Eight horticultural traits such as germination percentage (GP), germination vigor (GV), seedling length (SL), plant high (PH), seedling stem diameter (SSD), plant stem diameter (PSD), the average number of seeds per fruit (ANSF), plant type (PT), and marker-assisted selection (MAS) were used in our study for selection of tomato *S. l* (Table 1). These plant growth characteristics were an important indicator for commercial breeding. The ANSF was affected by fruit setting and fruit phenotype. In addition, tomato seed yield and quality are largely determined by the variety chosen for seed production [50]. According to Patwary et al. [51], the number of seeds per fruit varied from 26.0 to 107.70 in the winter to 4.02 to 49.39 in the summer. Tomato fruit set is best around 17–18 °C at night and 20–25.6 °C during the day [52,53]. Given that the maternal parent decides the quantity of ovules, supplies resources to the new embryo, and develops the seed coat, these findings are not surprising [54]. The germination percentages and the seed germination vigor were influenced by several factors, including the genetic constitution, mother plant environment and nutrition, harvest maturity, seed weight and size, mechanical integrity, degradation and ageing, and infections [55]. Additionally, disease resistance was selected as a single resistance and a combination of multiple resistances by molecular markers. Marker-assisted selection (MAS), which permits the selection of a single resistance gene or a combination of many resistance genes, has been widely and successfully used in tomato breeding projects, particularly for disease resistance [56,57].

Six horticultural traits, germination percentage (GP), germination vigor (GV), seedling length (SL), seedling stem diameter (SSD), plant stem diameter (PSD), average number of seeds per fruit (ANSF), and marker-assisted selection (MAS), were used in our study for the selection of tomato *S. h* (Table 2). In contrast, Ibrahim et al. [31], reported that seed germination rates are low, seed homogeneity is poor, and seed dormancy is high in wild species. The experiment revealed that seed germination of *S. h* was strong (Table 2), seed homogeneity was strong, and seed dormancy was low (data not shown). In addition, wild species are valuable sources of disease resistance and agronomic features in breeding efforts [58]. Earlier studies showed that *S. h* contain disease-resistance genes [59,60,61], pest resistance [62,63,64], cold tolerance, and quality traits [65,66] in some of these genes [67]. Additionally, Peralta et al. [2] found that the species is extremely vigorous, with a big spreading habit and a corolla up to 5 cm. This high vigor may be a major reason for its success in rootstock hybrids. In contrast, Huarachi Morejon et al. [68] reported that seed germination can be predicted by the genetic distance between female and male parents; however, some wide crossings can perform as well as or better than crosses with small-genetic-distance parents. The experiment revealed the seed germination percentage and germination vigor of intraspecific hybrids (*S. l* × *S. l*) were better than interspecific hybrids (*S. l* × *S. h*) (Table 4). In this study, these two lines (JTS33-3 and JTS35-4) were high GP and strong GV and the five wild lines (SN-42, SN-06, SN-08, SN-20 and SN-33) were high GP and strong GV. However, after crossing these two lines, the result showed that the seed germination of intraspecific hybrids was better than interspecific hybrids (Table 4). Horticultural traits such as plant height (PH), internode length (IL) and stem girth were used in this study at 60 DAT. Furthermore, these traits of interspecific hybrids were non-significantly compared to Maxifort, and these traits were better than intraspecific hybrids (Table 4). Plant height and stem girth are usually strong indicators of plant vitality, which can lead to higher yields. It is important to understand the relationship between plant characteristics, growth parameters, and yield. The height of the tomato plant and the diameter of the fruit have a strong positive correlation [69]. Plant height has also been found to have a substantial positive relationship with leaf metrics such as the number of leaves, leaf area, and leaf area index, as well as the number of branches [70]. The most frequent tomato rootstocks are tomato hybrids (intraspecific hybrids) and interspecific hybrids [25]. Interspecific hybrids are more vigorous and usually produce high-quality rootstocks with a large genetic diversity [71]. The stem diameter of intraspecific hybrids and interspecific hybrids was non-significantly different under the same condition (Table 4). Thus, the shortest stem diameter could be related to inadequate mineral, water, and photosynthetic transport from the earth to the plant [72]. This study indicated that quantifiable morphological differences exist between intraspecific hybrids and interspecific hybrids of root systems (Table 4 and Figure 4). In addition, Oztekin et al. [73] reported that two commercial rootstocks found variations in root density but not in average root diameter when it came to tomato rootstock root systems. Except for total root length, the root system morphology in tomato rootstocks varies by cultivar and is consistent through time. These distinctions could be used to classify cultivars for their suitability for use in certain growing situations, as well as to explain why specific rootstocks produce better growth and productivity [74]. The root system is a critical part of plant growth because it plays important functions in absorbing water and nutrients as well as a mechanical support and a storage organ as a barrier against pathogens [75,76]. *S. l* × *S. h* F_1_ hybrids with multiple resistance to soil-borne diseases are the most frequent commercial rootstocks. However, the genetic potential of *Solanum* spp. for rootstock development has yet to be completely realized.

In conclusion, tomato rootstock with multiple resistances and tolerances to biotic and abiotic stresses are required in order to justify the extra cost added in the production. At the same time, it is important to obtain high rootstock seed quality based on high germination and vigor. Screening multiple inbred lines crossed with multiple wild relatives can help to achieve these goals. The production of seeds is a complex interaction of genetics and environmental factors.

## Figures and Tables

**Table 1:** List of horticultural traits and gene/loci of *Solanum lycopersicum*.

No	Line	Horticultural Traits								Gene/Loci													
	Name	Germination		SL	PH	SSD	PSD	ANSF	Plant Type	Soil-Borne Pathogens							Vector-Borne					Air-Borne	
				(cm)	(cm)	(mm)	(mm)																
		GP (%)	GV	30 (DAS)	60 (DAT)	30 (DAS)	60 (DAT)	ea	ID/D	I3	Ve2	J3	Py1	Mi23	Bw6	Bw12	Sw5	Tm2a	Ty1	Ty2	Ph3	Sm-565	Cf9
										(F)	(F)	(F)	(F)	(N)	(B)	(B)	(V)	(V)	(V)	(V)	(F)	(F)	(F)
1	JTS01-3	57	5	14	148	4.3	14.75	22	D	**S**	**S**	**R**	**S**	**R**	**R**	**R**	**S**	**R**	**S**	**S**	**S**	**R**	**S**
2	JTS05-2	92	5	13	141	4.76	14.94	21	ID	**S**	**R**	**S**	**S**	**R**	**S**	**R**	**S**	**R**	**S**	**S**	**S**	**R**	**R**
3	JTS07-2	80	7	21	289	4.35	20.14	54	ID	**S**	**S**	**R**	**S**	**R**	**R**	**R**	**S**	**R**	**S**	**S**	**R**	**S**	**S**
4	JTS09-4	99	9	21	172	4.77	16.84	25	ID	**S**	**R**	**R**	**R**	**R**	**R**	**R**	**S**	**R**	**S**	**S**	**R**	**S**	**S**
5	JTS11-4	97	7	22	149	4.22	14.82	38	D	**S**	**S**	**R**	**R**	**R**	**R**	**R**	**S**	**R**	**S**	**S**	**S**	**S**	**S**
6	JTS16-3	93	5	14	137	3.98	14.92	36	D	**S**	**S**	**S**	**S**	**R**	**S**	**R**	**S**	**R**	**S**	**S**	**S**	**S**	**S**
7	JTS35-3	94	9	17	168	4.44	16.92	29	ID	**S**	**S**	**R**	**S**	**S**	**R**	**R**	**S**	**R**	**S**	**S**	**S**	**R**	**S**
8	JTS37-3	72	3	18	178	4.09	17.35	5	ID	**S**	**R**	**R**	**R**	**R**	**R**	**R**	**S**	**R**	**S**	**S**	**S**	**S**	**S**
9	JTS21-3	85	5	24	135	4.19	14.05	36	D	**R**	**R**	**R**	**S**	**R**	**R**	**R**	**S**	**R**	**S**	**S**	**S**	**R**	**S**
10	JTS25-4	74	3	22	142	4.21	13.81	44	D	**R**	**S**	**R**	**R**	**R**	**R**	**R**	**S**	**R**	**S**	**S**	**S**	**R**	**S**
11	JTS25-5	86	7	13	179	3.96	16.83	29	ID	**R**	**S**	**H**	**R**	**R**	**S**	**R**	**S**	**R**	**S**	**S**	**S**	**R**	**S**
12	JTS26-3	93	9	17	135	5.01	14.73	33	D	**S**	**S**	**R**	**R**	**R**	**R**	**R**	**S**	**R**	**S**	**S**	**S**	**R**	**S**
13	JTS27-2	31	3	19	139	3.98	13.98	25	D	**S**	**S**	**R**	**R**	**R**	**R**	**R**	**S**	**R**	**S**	**S**	**S**	**R**	**S**
14	JTS28-4	79	5	18	253	4.05	19.05	31	ID	**S**	**R**	**R**	**R**	**S**	**R**	**R**	**S**	**R**	**S**	**S**	**S**	**R**	**S**
15	JTS33-3	85	5	23	275	4.14	18.85	66	ID	**R**	**R**	**R**	**R**	**R**	**R**	**R**	**S**	**R**	**S**	**S**	**S**	**S**	**R**
16	JTS35-4	97	9	24	283	4.27	18.91	33	ID	**R**	**R**	**R**	**R**	**R**	**R**	**R**	**S**	**R**	**S**	**S**	**S**	**S**	**R**
17	JTS37-4	85	5	17	149	4.06	16.85	38	ID	**S**	**R**	**R**	**R**	**R**	**R**	**R**	**S**	**S**	**S**	**S**	**S**	**S**	**S**
18	JTS01-1	35	1	19	142	4.26	14.95	4	D	**R**	**R**	**R**	**S**	**R**	**R**	**R**	**S**	**R**	**S**	**S**	**S**	**R**	**S**
19	JTS01-2	48	1	17	147	4.09	14.72	11	D	**R**	**R**	**R**	**S**	**R**	**H**	**R**	**S**	**R**	**S**	**S**	**S**	**R**	**S**
20	JTS02-1	83	3	18	189	5.45	17.54	25	ID	**S**	**R**	**R**	**S**	**R**	**R**	**R**	**S**	**R**	**S**	**S**	**S**	**S**	**S**
21	JTS02-2	42	1	17	192	5.11	19.21	12	ID	**S**	**R**	**R**	**S**	**R**	**R**	**R**	**S**	**R**	**S**	**S**	**S**	**S**	**S**
22	JTS03-1	70	1	17	164	4.15	16.69	15	D	**S**	**H**	**R**	**S**	**R**	**R**	**R**	**S**	**R**	**S**	**S**	**S**	**R**	**S**
23	JTS03-2	94	5	15	134	3.98	13.57	8	D	**S**	**R**	**R**	**S**	**R**	**R**	**R**	**S**	**R**	**S**	**S**	**S**	**R**	**S**
24	JTS04-1	76	3	16	129	3.75	13.91	40	D	**H**	**R**	**R**	**S**	**R**	**R**	**R**	**S**	**R**	**S**	**S**	**S**	**R**	**S**
25	JTS04-2	57	5	20	132	4.02	13.85	35	D	**R**	**R**	**R**	**S**	**R**	**R**	**R**	**S**	**R**	**S**	**S**	**S**	**R**	**S**
26	JTS05-1	76	5	13	147	3.98	14.9	25	D	**R**	**R**	**H**	**S**	**R**	**S**	**R**	**S**	**R**	**S**	**S**	**S**	**R**	**S**
27	JTS06-1	95	7	18	148	4.27	14.98	25	D	**S**	**R**	**R**	**S**	**R**	**R**	**R**	**S**	**R**	**S**	**S**	**S**	**R**	**S**
28	JTS06-2	98	9	18	128	4.18	13.89	22	D	**S**	**R**	**R**	**S**	**H**	**H**	**R**	**S**	**R**	**S**	**S**	**S**	**R**	**S**
29	JTS06-3	95	9	18	134	4.32	13.28	15	D	**S**	**R**	**R**	**S**	**R**	**S**	**R**	**S**	**R**	**S**	**S**	**S**	**R**	**S**
30	JTS06-4	95	9	12	149	3.9	14.98	14	D	**H**	**R**	**R**	**S**	**R**	**R**	**R**	**S**	**R**	**S**	**S**	**S**	**R**	**S**
31	JTS07-1	67	5	19	154	3.95	15.02	21	ID	**S**	**R**	**R**	**S**	**R**	**S**	**R**	**S**	**R**	**S**	**S**	**R**	**S**	**S**
32	JTS08-1	72	5	15	152	4.31	15.64	26	ID	**R**	**H**	**R**	**R**	**R**	**R**	**R**	**S**	**R**	**S**	**S**	**S**	**R**	**R**
33	JTS08-2	81	5	14	168	4.15	15.89	17	ID	**R**	**R**	**R**	**R**	**R**	**R**	**R**	**S**	**R**	**S**	**S**	**S**	**R**	**R**
34	JTS08-3	90	5	14	158	4.35	16.01	22	ID	**R**	**R**	**R**	**R**	**R**	**R**	**R**	**S**	**R**	**S**	**S**	**S**	**R**	**R**
35	JTS09-1	35	1	15	168	4.71	16.05	6	ID	**R**	**R**	**R**	**R**	**R**	**R**	**R**	**S**	**R**	**S**	**S**	**R**	**S**	**S**
36	JTS09-2	81	3	17	175	4.52	16.85	2	ID	**R**	**R**	**R**	**R**	**R**	**R**	**R**	**S**	**R**	**S**	**S**	**R**	**S**	**S**
37	JTS09-3	43	1	18	198	4.76	19.58	4	ID	**R**	**R**	**R**	**R**	**R**	**R**	**R**	**S**	**R**	**S**	**S**	**R**	**S**	**S**
38	JTS10-1	95	5	17	230	3.97	19.02	17	ID	**S**	**R**	**R**	**R**	**R**	**R**	**R**	**S**	**R**	**S**	**S**	**R**	**S**	**S**
39	JTS10-2	91	7	17	192	4.11	17.85	11	ID	**S**	**R**	**R**	**R**	**R**	**R**	**R**	**S**	**R**	**S**	**S**	**R**	**S**	**S**
40	JTS11-1	62	3	19	178	4.15	18.92	33	D	**H**	**S**	**R**	**R**	**R**	**R**	**R**	**S**	**R**	**S**	**S**	**S**	**R**	**S**
41	JTS11-2	82	3	21	181	4.84	18.08	19	D	**R**	**S**	**R**	**R**	**R**	**R**	**R**	**S**	**R**	**S**	**S**	**S**	**R**	**S**
42	JTS11-3	65	3	20	148	4.4	14.58	13	D	**R**	**S**	**R**	**R**	**R**	**R**	**R**	**S**	**R**	**S**	**S**	**S**	**R**	**S**
43	JTS12-1	98	7	19	145	3.98	13.98	17	ID	**S**	**R**	**R**	**R**	**R**	**R**	**R**	**S**	**R**	**S**	**S**	**S**	**R**	**S**
44	JTS12-2	99	9	18	124	4.61	13.24	15	ID	**S**	**R**	**R**	**R**	**R**	**R**	**R**	**S**	**R**	**S**	**S**	**S**	**R**	**S**
45	JTS13-1	96	9	22	201	4.98	17.89	34	ID	**R**	**R**	**R**	**R**	**R**	**R**	**R**	**S**	**R**	**S**	**S**	**S**	**S**	**S**
46	JTS13-2	99	9	23	258	5.23	19.15	26	ID	**R**	**R**	**R**	**R**	**R**	**R**	**R**	**S**	**R**	**S**	**S**	**S**	**S**	**S**
47	JTS13-3	86	7	24	178	4.12	15.98	13	ID	**R**	**R**	**R**	**R**	**R**	**R**	**R**	**S**	**R**	**S**	**S**	**S**	**S**	**S**
48	JTS14-1	98	9	21	165	4.01	16.35	30	ID	**R**	**H**	**R**	**R**	**R**	**R**	**R**	**S**	**R**	**S**	**S**	**S**	**S**	**S**
49	JTS14-2	57	5	24	175	3.93	15.21	28	ID	**R**	**R**	**R**	**R**	**R**	**R**	**R**	**S**	**R**	**S**	**S**	**S**	**S**	**S**
50	JTS14-3	98	9	22	221	3.88	19.07	29	ID	**R**	**R**	**R**	**R**	**R**	**R**	**R**	**S**	**R**	**S**	**S**	**S**	**S**	**S**
51	JTS15-1	67	7	23	212	4.11	18.54	2	ID	**S**	**R**	**R**	**R**	**R**	**R**	**R**	**S**	**R**	**S**	**S**	**S**	**S**	**S**
52	JTS16-1	95	7	14	165	3.95	15.89	37	ID	**S**	**S**	**H**	**S**	**R**	**R**	**R**	**S**	**R**	**S**	**S**	**S**	**R**	**S**
53	JTS16-2	98	7	16	175	3.85	17.68	45	ID	**S**	**R**	**H**	**S**	**R**	**R**	**R**	**S**	**R**	**S**	**S**	**S**	**R**	**S**
54	JTS17-1	96	9	16	185	4.15	17.85	13	ID	**S**	**R**	**S**	**S**	**H**	**R**	**R**	**S**	**S**	**S**	**S**	**S**	**R**	**S**
55	JTS17-2	77	7	17	198	3.91	18.95	21	ID	**S**	**R**	**S**	**S**	**H**	**R**	**R**	**S**	**S**	**S**	**S**	**S**	**R**	**S**
56	JTS18-1	90	7	15	162	3.89	17.21	17	ID	**S**	**S**	**H**	**S**	**R**	**R**	**R**	**S**	**R**	**S**	**S**	**S**	**S**	**S**
57	JTS18-2	95	9	14	173	3.79	16.85	37	ID	**S**	**R**	**H**	**S**	**R**	**R**	**R**	**S**	**R**	**S**	**S**	**S**	**R**	**S**
58	JTS19-1	93	9	18	149	4.08	13.86	8	D	**S**	**S**	**H**	**S**	**R**	**R**	**R**	**S**	**R**	**S**	**S**	**S**	**S**	**S**
59	JTS19-2	78	7	17	142	3.97	13.55	15	D	**S**	**S**	**H**	**S**	**R**	**R**	**R**	**S**	**R**	**S**	**S**	**S**	**S**	**S**
60	JTS19-3	91	9	16	135	4.15	12.38	25	D	**S**	**S**	**H**	**S**	**R**	**R**	**R**	**S**	**R**	**S**	**S**	**S**	**S**	**S**
61	JTS20-1	95	7	20	198	3.96	18.25	64	ID	**S**	**R**	**H**	**S**	**R**	**R**	**R**	**S**	**H**	**S**	**S**	**S**	**S**	**S**
62	JTS20-2	89	7	17	215	3.89	18.01	12	ID	**S**	**R**	**H**	**S**	**R**	**R**	**R**	**S**	**H**	**S**	**S**	**H**	**S**	**S**
63	JTS21-1	68	5	26	138	3.85	13.89	18	D	**R**	**R**	**R**	**S**	**R**	**R**	**R**	**S**	**R**	**S**	**S**	**S**	**R**	**S**
64	JTS21-2	72	7	20	141	3.91	12.89	24	D	**S**	**R**	**R**	**S**	**R**	**S**	**R**	**S**	**R**	**S**	**S**	**S**	**R**	**S**
65	JTS23-1	90	9	21	148	3.52	14.25	27	ID	**S**	**R**	**R**	**S**	**R**	**R**	**R**	**S**	**R**	**S**	**S**	**S**	**S**	**S**
66	JTS23-2	92	9	21	169	3.8	16.08	27	ID	**S**	**R**	**R**	**S**	**R**	**R**	**R**	**S**	**R**	**S**	**S**	**S**	**R**	**S**
67	JTS23-3	93	7	20	179	4.01	16.23	12	ID	**S**	**R**	**R**	**S**	**R**	**H**	**R**	**S**	**R**	**S**	**S**	**S**	**S**	**S**
68	JTS24-1	96	9	23	181	4.45	18.25	33	ID	**S**	**R**	**R**	**S**	**R**	**R**	**R**	**S**	**R**	**S**	**S**	**R**	**S**	**R**
69	JTS24-2	99	9	22	175	4.11	18.01	40	ID	**S**	**R**	**R**	**S**	**R**	**S**	**H**	**S**	**R**	**S**	**S**	**R**	**S**	**R**
70	JTS24-3	95	7	20	182	4.09	15.89	25	ID	**S**	**R**	**R**	**R**	**R**	**R**	**R**	**S**	**R**	**S**	**S**	**H**	**S**	**R**
71	JTS25-1	92	7	18	168	4.02	16.65	8	ID	**R**	**S**	**H**	**R**	**R**	**S**	**R**	**S**	**R**	**S**	**S**	**S**	**R**	**S**
72	JTS25-2	95	7	20	181	4.26	17.98	21	ID	**R**	**R**	**H**	**R**	**R**	**S**	**R**	**S**	**R**	**S**	**S**	**S**	**R**	**S**
73	JTS25-3	90	7	19	192	4.82	17.55	14	ID	**R**	**R**	**R**	**S**	**R**	**S**	**R**	**S**	**R**	**S**	**S**	**S**	**S**	**S**
74	JTS26-1	76	5	20	187	4.2	17.35	38	ID	**S**	**R**	**R**	**S**	**R**	**R**	**R**	**S**	**R**	**S**	**S**	**S**	**S**	**S**
75	JTS26-2	94	7	21	205	3.83	16.98	39	ID	**S**	**R**	**R**	**S**	**R**	**R**	**R**	**S**	**R**	**S**	**S**	**S**	**S**	**S**
76	JTS27-1	90	5	18	181	4.08	16.85	5	ID	**S**	**S**	**S**	**S**	**R**	**R**	**H**	**S**	**R**	**S**	**S**	**S**	**R**	**S**
77	JTS28-1	97	9	22	178	4.72	16.91	50	ID	**S**	**R**	**R**	**S**	**R**	**S**	**R**	**S**	**R**	**S**	**S**	**S**	**R**	**S**
78	JTS28-2	95	9	23	189	4.34	18.25	26	ID	**S**	**R**	**R**	**S**	**R**	**S**	**R**	**S**	**R**	**S**	**S**	**S**	**R**	**S**
79	JTS28-3	78	7	21	208	4.31	18.21	10	ID	**S**	**R**	**R**	**S**	**H**	**R**	**R**	**S**	**R**	**S**	**S**	**S**	**R**	**S**
80	JTS29-1	95	9	20	191	3.87	17.02	33	ID	**S**	**R**	**S**	**S**	**R**	**S**	**R**	**S**	**R**	**S**	**S**	**S**	**R**	**S**
81	JTS29-2	85	7	16	195	4.05	16.52	36	ID	**S**	**R**	**S**	**S**	**R**	**S**	**R**	**S**	**R**	**S**	**S**	**S**	**R**	**S**
82	JTS29-3	92	9	18	125	3.91	13.45	25	D	**S**	**R**	**S**	**S**	**R**	**R**	**R**	**S**	**S**	**S**	**S**	**S**	**R**	**S**
83	JTS30-1	86	9	17	138	4.39	13.86	44	D	**S**	**R**	**S**	**S**	**R**	**S**	**R**	**S**	**S**	**S**	**S**	**S**	**R**	**S**
84	JTS30-2	95	9	18	148	4.11	14.78	27	D	**S**	**R**	**S**	**S**	**R**	**H**	**R**	**S**	**S**	**S**	**S**	**S**	**R**	**S**
85	JTS32-1	80	5	19	182	3.92	16.25	14	ID	**S**	**S**	**R**	**S**	**R**	**R**	**R**	**S**	**R**	**S**	**S**	**S**	**S**	**S**
86	JTS32-2	95	5	15	168	3.71	15.85	75	ID	**S**	**R**	**R**	**S**	**R**	**R**	**R**	**S**	**R**	**S**	**S**	**S**	**R**	**S**
87	JTS33-1	91	7	24	186	3.69	16.35	18	ID	**S**	**S**	**R**	**S**	**R**	**R**	**R**	**S**	**R**	**S**	**S**	**S**	**R**	**S**
88	JTS33-2	89	7	25	181	3.89	15.21	28	ID	**S**	**R**	**R**	**S**	**R**	**R**	**R**	**S**	**R**	**S**	**S**	**S**	**R**	**S**
89	JTS34-1	78	7	22	198	3.98	18.21	25	ID	**S**	**S**	**H**	**S**	**H**	**R**	**R**	**S**	**H**	**S**	**S**	**S**	**R**	**R**
90	JTS35-1	98	9	21	178	4.18	15.89	29	ID	**S**	**R**	**H**	**S**	**R**	**R**	**R**	**S**	**R**	**S**	**S**	**S**	**R**	**S**
91	JTS35-2	99	9	24	195	3.49	16.23	25	ID	**S**	**R**	**S**	**S**	**S**	**S**	**R**	**S**	**S**	**S**	**S**	**S**	**R**	**S**
92	JTS36-1	82	7	16	197	3.91	16.25	8	ID	**S**	**S**	**H**	**S**	**H**	**H**	**R**	**S**	**R**	**S**	**S**	**S**	**R**	**S**
93	JTS36-2	90	9	18	215	3.82	15.89	5	ID	**S**	**S**	**R**	**S**	**R**	**R**	**R**	**S**	**R**	**S**	**S**	**S**	**R**	**S**
94	JTS37-1	86	7	19	201	3.92	16.02	50	ID	**S**	**R**	**R**	**R**	**R**	**R**	**R**	**S**	**S**	**S**	**S**	**S**	**S**	**S**
95	JTS37-2	78	5	20	235	3.95	15.89	23	ID	**S**	**R**	**R**	**R**	**R**	**R**	**R**	**S**	**S**	**S**	**S**	**S**	**S**	**S**
96	JTS38-1	93	5	19	208	4.28	15.98	14	ID	**S**	**R**	**H**	**S**	**R**	**S**	**R**	**S**	**R**	**S**	**S**	**S**	**S**	**S**
97	JTS39-1	74	5	15	161	4.02	18.91	28	ID	**S**	**R**	**S**	**S**	**R**	**S**	**R**	**S**	**S**	**S**	**S**	**S**	**R**	**S**
98	JTS39-2	51	3	17	159	4.73	19.05	43	ID	**S**	**R**	**S**	**S**	**R**	**S**	**S**	**S**	**S**	**S**	**S**	**S**	**R**	**S**
99	JTS40-1	84	5	15	158	3.85	17.21	9	D	**S**	**R**	**H**	**S**	**R**	**H**	**R**	**S**	**H**	**S**	**S**	**S**	**R**	**S**
100	JTS40-2	94	5	16	162	3.97	17.05	30	D	**S**	**R**	**H**	**S**	**R**	**S**	**R**	**S**	**H**	**S**	**S**	**S**	**S**	**S**
101	JTS42-1	95	5	14	173	3.82	16.86	20	ID	**S**	**R**	**R**	**S**	**R**	**S**	**R**	**S**	**R**	**S**	**S**	**S**	**R**	**R**

I3: Fusarium wilt, Ve2: Verticillium wilt, J3: Fusarium crown and root rot, py1: Corky root rot, Mi23: Root-Knot nematode, Bw6, Bw12: Bacterial wilt, Sw5: TSWV (Tomato Spotted wilt virus), Tm2a: ToMV (Tomato mosaic virus), Ty1, Ty2: TYLCV (Tomato yellow leaf curl virus), Ph3: Late blight, Sm-565: Gray leaf spot, Cf9: Leaf mold. ID: indeterminate, D: determinate, R: resistant, S: susceptible, H: heterozygous, B: bacteria, F: fungus, V: virus, N: nematode. SL: seedling length, PH: plant height, SSD: seedling stem diameter, PSD: plant stem diameter, ANSF: average number of seed per fruit, GP: Germination percentage, GV: germination vigor (note 1 = very weak, 2 = very weak to weak, 3 = weak, 4 = weak to medium, 5 = medium, 6 = medium to strong, 7 = strong, 8 = strong to very strong, 9 = very strong) [34].

**Table 2:** Lists horticultural traits and Gene/Loci of *Solanum habrochaites*.

No	Line	Horticultural Traits						Gene/Loci														
	Name	Germination		SL	SSD	PSD	ANSF	Soil-Borne Pathogens								Vector-Borne				Air-Borne		
				(Cm)	(mm)	(mm)																
		GP (%)	GV	30 (DAS)	30 (DAS)	60 (DAT)	ea	I2	I3	Ve2	J3	Py1	Mi23	Bw6	Bw12	Sw5	Tm2a	Ty1	Ty2	Ph3	Sm-565	Cf9
								(F)	(F)	(F)	(F)	(F)	(N)	(B)	(B)	(V)	(V)	(V)	(V)	(F)	(F)	(F)
1	SN-01	75	3	10	3.46	12.85	7	**⁃**	**S**	**S**	**R**	**S**	**⁃**	**S**	**S**	**R**	**S**	**S**	**R**	**R**	**R**	**⁃**
2	SN-02	76	7	10	3.14	12.56	17	**⁃**	**S**	**S**	**R**	**S**	**⁃**	**S**	**S**	**R**	**S**	**S**	**R**	**R**	**R**	**⁃**
3	SN-03	96	7	8	2.94	12.33	20	**⁃**	**S**	**S**	**R**	**S**	**⁃**	**S**	**S**	**R**	**S**	**S**	**R**	**R**	**R**	**⁃**
4	SN-04	78	3	10	2.74	12.95	15	**⁃**	**S**	**S**	**R**	**S**	**⁃**	**S**	**S**	**R**	**S**	**S**	**R**	**R**	**R**	**⁃**
5	SN-05	98	7	9	3.24	12.76	15	**⁃**	**S**	**S**	**R**	**S**	**⁃**	**S**	**S**	**R**	**S**	**S**	**R**	**R**	**R**	**⁃**
6	SN-06	99	5	10	3.21	12.91	7	**⁃**	**S**	**S**	**R**	**S**	**⁃**	**S**	**S**	**R**	**S**	**S**	**R**	**R**	**R**	**⁃**
7	SN-07	98	5	8	2.63	12.84	20	**⁃**	**S**	**S**	**R**	**S**	**⁃**	**S**	**S**	**R**	**S**	**S**	**R**	**R**	**R**	**⁃**
8	SN-08	99	5	9	2.69	12.85	10	**⁃**	**S**	**S**	**R**	**S**	**⁃**	**S**	**S**	**R**	**S**	**S**	**R**	**R**	**R**	**⁃**
9	SN-09	90	5	11	2.96	12.87	29	**⁃**	**S**	**S**	**R**	**S**	**⁃**	**S**	**S**	**R**	**S**	**S**	**R**	**R**	**R**	**⁃**
10	SN-10	86	3	7	2.29	12.55	8	**⁃**	**S**	**S**	**R**	**S**	**⁃**	**S**	**S**	**R**	**S**	**S**	**R**	**R**	**R**	**⁃**
11	SN-11	78	3	9	2.23	12.31	11	**⁃**	**S**	**S**	**R**	**S**	**⁃**	**S**	**S**	**R**	**S**	**S**	**R**	**R**	**R**	**⁃**
12	SN-12	85	7	9	2.48	12.34	33	**⁃**	**S**	**S**	**R**	**S**	**⁃**	**S**	**S**	**R**	**S**	**S**	**R**	**R**	**R**	**⁃**
13	SN-13	97	9	12	2.91	12.86	24	**⁃**	**S**	**S**	**R**	**S**	**⁃**	**S**	**S**	**R**	**S**	**S**	**R**	**R**	**R**	**⁃**
14	SN-14	99	5	10	3.07	14.85	6	**⁃**	**S**	**S**	**R**	**S**	**⁃**	**S**	**S**	**R**	**S**	**S**	**R**	**R**	**R**	**⁃**
15	SN-15	43	3	8	2.58	12.51	10	**⁃**	**S**	**S**	**R**	**S**	**⁃**	**S**	**S**	**R**	**S**	**S**	**R**	**R**	**R**	**⁃**
16	SN-16	76	5	10	3.07	12.75	9	**⁃**	**S**	**S**	**R**	**S**	**⁃**	**S**	**S**	**R**	**S**	**S**	**R**	**R**	**R**	**⁃**
17	SN-17	58	5	10	3.11	12.88	21	**⁃**	**S**	**S**	**R**	**S**	**⁃**	**S**	**S**	**R**	**S**	**S**	**R**	**R**	**R**	**⁃**
18	SN-18	52	5	9	2.71	12.45	17	**⁃**	**S**	**S**	**R**	**S**	**⁃**	**S**	**S**	**R**	**S**	**S**	**R**	**R**	**R**	**⁃**
19	SN-19	75	3	8	3.05	12.75	30	**⁃**	**S**	**S**	**R**	**S**	**⁃**	**S**	**S**	**R**	**S**	**S**	**R**	**R**	**R**	**⁃**
20	SN-20	98	7	10	3.06	12.91	14	**⁃**	**S**	**S**	**R**	**S**	**⁃**	**S**	**S**	**R**	**S**	**S**	**R**	**R**	**R**	**⁃**
21	SN-21	90	7	10	2.97	12.89	13	**⁃**	**S**	**S**	**R**	**S**	**⁃**	**S**	**S**	**R**	**S**	**S**	**R**	**R**	**R**	**⁃**
22	SN-22	89	7	13	3.61	13.09	11	**⁃**	**S**	**S**	**R**	**S**	**⁃**	**S**	**S**	**R**	**S**	**S**	**R**	**R**	**R**	**⁃**
23	SN-23	90	9	13	3.62	13.06	12	**⁃**	**S**	**S**	**R**	**S**	**⁃**	**S**	**S**	**R**	**S**	**S**	**R**	**R**	**R**	**⁃**
24	SN-24	99	9	13	3.6	13.05	13	**⁃**	**S**	**S**	**R**	**S**	**⁃**	**S**	**S**	**R**	**S**	**S**	**R**	**R**	**R**	**⁃**
25	SN-25	98	9	14	3.13	12.86	16	**⁃**	**S**	**S**	**R**	**S**	**⁃**	**S**	**S**	**R**	**S**	**S**	**R**	**R**	**R**	**⁃**
26	SN-26	98	5	13	2.85	12.35	14	**⁃**	**S**	**S**	**R**	**S**	**⁃**	**S**	**S**	**R**	**S**	**S**	**R**	**R**	**R**	**⁃**
27	SN-27	91	7	14	2.63	12.54	11	**⁃**	**S**	**S**	**R**	**S**	**⁃**	**S**	**S**	**R**	**S**	**S**	**R**	**R**	**R**	**⁃**
28	SN-28	81	5	12	3.04	12.85	14	**⁃**	**S**	**S**	**R**	**S**	**⁃**	**S**	**S**	**R**	**S**	**S**	**R**	**R**	**R**	**⁃**
29	SN-29	78	5	14	3.32	12.99	13	**⁃**	**S**	**S**	**R**	**S**	**⁃**	**S**	**S**	**R**	**S**	**S**	**R**	**R**	**R**	**⁃**
30	SN-30	95	7	11	2.71	12.86	16	**⁃**	**S**	**S**	**R**	**S**	**⁃**	**S**	**S**	**R**	**S**	**S**	**R**	**R**	**R**	**⁃**
31	SN-31	91	7	16	3.66	12.98	13	**⁃**	**S**	**S**	**R**	**S**	**⁃**	**S**	**S**	**R**	**S**	**S**	**R**	**R**	**R**	**⁃**
32	SN-32	94	9	13	3.36	13.03	9	**⁃**	**S**	**S**	**R**	**S**	**⁃**	**S**	**S**	**R**	**S**	**S**	**R**	**R**	**R**	**⁃**
33	SN-33	81	9	15	3.62	13.02	17	**⁃**	**S**	**S**	**R**	**S**	**⁃**	**S**	**S**	**R**	**S**	**S**	**R**	**R**	**R**	**⁃**
34	SN-34	99	7	11	3.15	13.05	11	**⁃**	**S**	**S**	**R**	**S**	**⁃**	**S**	**S**	**R**	**S**	**S**	**R**	**R**	**R**	**⁃**
35	SN-35	95	9	13	3.33	13.01	13	**⁃**	**S**	**S**	**R**	**S**	**⁃**	**S**	**S**	**R**	**S**	**S**	**R**	**R**	**R**	**⁃**
36	SN-36	99	7	11	2.95	12.78	12	**⁃**	**S**	**S**	**R**	**S**	**⁃**	**S**	**S**	**R**	**S**	**S**	**R**	**R**	**R**	**⁃**
37	SN-37	95	9	10	2.78	12.8	6	**⁃**	**S**	**S**	**R**	**S**	**⁃**	**S**	**S**	**R**	**S**	**S**	**R**	**R**	**R**	**⁃**
38	SN-38	52	3	10	2.8	12.75	7	**⁃**	**S**	**S**	**R**	**S**	**⁃**	**S**	**S**	**R**	**S**	**S**	**R**	**R**	**R**	**⁃**
39	SN-39	75	3	14	3.04	12.84	16	**⁃**	**S**	**S**	**R**	**S**	**⁃**	**S**	**S**	**R**	**S**	**S**	**R**	**R**	**R**	**⁃**
40	SN-40	90	5	14	2.98	12.84	11	**⁃**	**S**	**S**	**R**	**S**	**⁃**	**S**	**S**	**R**	**S**	**S**	**R**	**R**	**R**	**⁃**
41	SN-41	91	5	13	3.33	12.89	13	**⁃**	**S**	**S**	**R**	**S**	**⁃**	**S**	**S**	**R**	**S**	**S**	**R**	**R**	**R**	**⁃**
42	SN-42	98	7	14	2.8	13.02	12	**⁃**	**S**	**S**	**R**	**S**	**⁃**	**S**	**S**	**R**	**S**	**S**	**R**	**R**	**R**	**⁃**

I2, I3: Fusarium wilt, Ve2: Verticillium wilt, J3: Fusarium crown and root rot, py1: Corky root rot, Mi23: Root-Knot nematode, Bw6, Bw12: Bacterial wilt, Sw5: TSWV (Tomato Spotted wilt virus), Tm2a: ToMV (Tomato mosaic virus), Ty1, Ty2: TYLCV (Tomato yellow leaf curl virus), Ph3: Late blight, Sm-565: Gray leaf spot, Cf9: Leaf mold. R: resistant, S: susceptible, B: bacteria, F: fungus, V: virus, N: nematode, (⁃) or N/A: not amplified. SL: seedling length, SSD: seedling stem diameter, PSD: plant stem diameter, ASNF: average seed of number per fruit, GP: germination percentage, GV: Germination vigor (note 1 ₌ very weak, 2 ₌ very weak to weak, 3 ₌ weak, 4 ₌ weak to medium, 5 ₌ medium, 6 ₌ medium to strong, 7 ₌ strong, 8 ₌ strong to very strong, 9 ₌ very strong) [34].

**Table 3:** List of gene/loci of hybrid new combination; interspecific (*S. l* × *S. h*), intraspecific (*S. l* × *S. l*), and commercial rootstock (Maxifort).

No	Name	Soil-Borne Pathogens								Vector-Borne				Air-Borne		
		I2	I3	Ve2	J3	Py1	Mi23	Bw6	Bw12	Sw5	Tm2a	Ty1	Ty2	Ph3	Sm-565	Cf9
		(F)	(F)	(F)	(F)	(F)	(N)	(B)	(B)	(V)	(V)	(V)	(V)	(F)	(F)	(F)
	Interspecific rootstock (*S. l* × *S. h*)															
1	JTS01-3 × SN-42	**S**	**S**	**S**	**R**	**S**	**R**	**H**	**H**	**H**	**H**	**S**	**H**	**H**	**R**	**S**
2	JTS05-2 × SN-42	**S**	**S**	**S**	**R**	**S**	**R**	**H**	**H**	**H**	**H**	**S**	**H**	**H**	**R**	**S**
3	JTS07-2 × SN-42	**H**	**S**	**S**	**R**	**S**	**R**	**H**	**H**	**H**	**H**	**S**	**H**	**R**	**H**	**S**
4	JTS09-4 × SN-42	**H**	**S**	**R**	**R**	**R**	**R**	**H**	**H**	**H**	**H**	**S**	**H**	**R**	**H**	**S**
5	JTS11-4 × SN-42	**S**	**S**	**S**	**R**	**R**	**R**	**H**	**H**	**H**	**H**	**S**	**H**	**H**	**H**	**S**
6	JTS16-3 × SN-42	**S**	**S**	**S**	**H**	**S**	**R**	**S**	**H**	**H**	**H**	**S**	**H**	**H**	**H**	**S**
7	JTS21-3 × SN-42	**H**	**H**	**R**	**R**	**S**	**R**	**H**	**H**	**H**	**H**	**S**	**H**	**H**	**R**	**S**
8	JTS25-4 × SN-42	**H**	**H**	**S**	**R**	**R**	**R**	**H**	**H**	**H**	**H**	**S**	**H**	**H**	**R**	**S**
9	JTS25-5 × SN-42	**H**	**H**	**S**	**H**	**R**	**R**	**S**	**H**	**H**	**H**	**S**	**H**	**H**	**R**	**S**
10	JTS27-2 × SN-42	**H**	**S**	**S**	**R**	**R**	**R**	**H**	**H**	**H**	**H**	**S**	**H**	**H**	**R**	**S**
11	JTS28-4 × SN-42	**S**	**S**	**R**	**R**	**R**	**S**	**H**	**H**	**H**	**H**	**S**	**H**	**H**	**R**	**S**
12	JTS35-4 × SN-42	**H**	**H**	**R**	**R**	**R**	**R**	**H**	**H**	**H**	**H**	**S**	**H**	**H**	**H**	**R**
13	JTS37-4 × SN-42	**H**	**S**	**R**	**R**	**R**	**R**	**H**	**H**	**H**	**S**	**S**	**H**	**H**	**H**	**S**
14	JTS01-3 × SN-06	**H**	**S**	**S**	**R**	**S**	**R**	**H**	**H**	**H**	**H**	**S**	**H**	**H**	**R**	**S**
15	JTS05-2 × SN-06	**H**	**H**	**R**	**H**	**S**	**R**	**S**	**H**	**H**	**H**	**S**	**H**	**H**	**R**	**S**
16	JTS09-4 × SN-06	**H**	**S**	**H**	**R**	**R**	**R**	**H**	**H**	**H**	**H**	**S**	**H**	**R**	**H**	**S**
17	JTS11-4 × SN-06	**S**	**S**	**S**	**R**	**R**	**R**	**H**	**H**	**H**	**H**	**S**	**H**	**H**	**H**	**S**
18	JTS16-3 × SN-06	**S**	**S**	**S**	**H**	**S**	**R**	**S**	**H**	**H**	**H**	**S**	**H**	**H**	**H**	**S**
19	JTS35-3 × SN-06	**H**	**S**	**S**	**R**	**S**	**S**	**H**	**H**	**H**	**H**	**S**	**H**	**H**	**R**	**S**
20	JTS37-3 × SN-06	**H**	**S**	**R**	**R**	**R**	**R**	**H**	**H**	**H**	**H**	**S**	**H**	**H**	**H**	**S**
21	JTS21-3 × SN-06	**H**	**H**	**R**	**R**	**S**	**R**	**H**	**H**	**H**	**H**	**S**	**H**	**H**	**R**	**S**
22	JTS25-5 × SN-06	**H**	**H**	**S**	**H**	**R**	**R**	**S**	**H**	**H**	**H**	**S**	**H**	**H**	**R**	**S**
23	JTS26-3 × SN-06	**H**	**S**	**S**	**R**	**R**	**R**	**H**	**H**	**H**	**H**	**S**	**H**	**H**	**R**	**S**
24	JTS28-4 × SN-06	**S**	**S**	**R**	**R**	**R**	**S**	**H**	**H**	**H**	**H**	**S**	**H**	**H**	**R**	**S**
25	JTS33-3 × SN-06	**H**	**H**	**H**	**R**	**R**	**R**	**H**	**H**	**H**	**H**	**S**	**H**	**H**	**H**	**S**
26	JTS35-4 × SN-06	**H**	**H**	**H**	**R**	**R**	**R**	**H**	**H**	**H**	**H**	**S**	**H**	**H**	**H**	**R**
27	JTS01-3 × SN-08	**H**	**S**	**S**	**R**	**S**	**R**	**H**	**H**	**H**	**H**	**S**	**H**	**H**	**R**	**S**
28	JTS05-2 × SN-08	**H**	**S**	**R**	**H**	**S**	**R**	**S**	**H**	**H**	**H**	**S**	**H**	**H**	**R**	**S**
29	JTS07-2 × SN-08	**H**	**S**	**S**	**R**	**S**	**R**	**H**	**H**	**H**	**H**	**S**	**H**	**R**	**H**	**S**
30	JTS09-4 × SN-08	**H**	**S**	**R**	**R**	**R**	**R**	**H**	**H**	**H**	**H**	**S**	**H**	**R**	**H**	**S**
31	JTS11-4 × SN-08	**H**	**S**	**S**	**R**	**R**	**R**	**H**	**H**	**H**	**H**	**S**	**H**	**H**	**H**	**S**
32	JTS16-3 × SN-08	**H**	**S**	**S**	**H**	**S**	**R**	**S**	**H**	**H**	**H**	**S**	**H**	**H**	**H**	**S**
33	JTS35-3 × SN-08	**S**	**S**	**S**	**R**	**S**	**S**	**H**	**H**	**H**	**H**	**S**	**H**	**H**	**R**	**S**
34	JTS37-3 × SN-08	**H**	**S**	**R**	**R**	**R**	**R**	**H**	**H**	**H**	**H**	**S**	**H**	**H**	**H**	**S**
35	JTS21-3 × SN-08	**H**	**H**	**R**	**R**	**S**	**R**	**H**	**H**	**H**	**H**	**S**	**H**	**H**	**R**	**S**
36	JTS25-4 × SN-08	**H**	**S**	**S**	**R**	**R**	**R**	**H**	**H**	**H**	**H**	**S**	**H**	**H**	**R**	**S**
37	JTS25-5 × SN-08	**H**	**H**	**S**	**R**	**R**	**R**	**S**	**H**	**H**	**H**	**S**	**H**	**H**	**R**	**S**
38	JTS26-3 × SN-08	**H**	**S**	**S**	**R**	**R**	**R**	**H**	**H**	**H**	**H**	**S**	**H**	**H**	**R**	**S**
39	JTS27-2 × SN-08	**H**	**S**	**S**	**R**	**R**	**R**	**H**	**H**	**H**	**H**	**S**	**H**	**H**	**R**	**S**
40	JTS28-4 × SN-08	**H**	**S**	**R**	**R**	**R**	**S**	**H**	**H**	**H**	**H**	**S**	**H**	**H**	**R**	**S**
41	JTS33-3 × SN-08	**H**	**H**	**R**	**R**	**R**	**R**	**H**	**H**	**H**	**H**	**S**	**H**	**H**	**H**	**R**
42	JTS35-4 × SN-08	**H**	**H**	**R**	**R**	**R**	**R**	**H**	**H**	**H**	**H**	**S**	**H**	**H**	**H**	**R**
43	JTS01-3 × SN-20	**S**	**S**	**S**	**R**	**S**	**R**	**H**	**H**	**H**	**H**	**S**	**H**	**H**	**R**	**S**
44	JTS05-2 × SN-20	**H**	**S**	**R**	**H**	**S**	**R**	**S**	**H**	**H**	**H**	**S**	**H**	**H**	**R**	**S**
45	JTS07-2 × SN-20	**H**	**S**	**S**	**R**	**S**	**R**	**H**	**H**	**H**	**H**	**S**	**H**	**R**	**H**	**S**
46	JTS11-4 × SN-20	**S**	**S**	**S**	**R**	**R**	**R**	**H**	**H**	**H**	**H**	**S**	**H**	**H**	**H**	**S**
47	JTS16-3 × SN-20	**S**	**S**	**S**	**H**	**S**	**R**	**S**	**H**	**H**	**H**	**S**	**H**	**H**	**H**	**S**
48	JTS37-3 × SN-20	**H**	**S**	**H**	**R**	**R**	**R**	**H**	**H**	**H**	**H**	**S**	**H**	**H**	**H**	**S**
49	JTS21-3 × SN-20	**H**	**H**	**R**	**R**	**S**	**R**	**H**	**H**	**H**	**H**	**S**	**H**	**H**	**R**	**S**
50	JTS25-4 × SN-20	**H**	**S**	**S**	**R**	**R**	**R**	**H**	**H**	**H**	**H**	**S**	**H**	**H**	**R**	**S**
51	JTS25-5 × SN-20	**H**	**S**	**S**	**H**	**R**	**R**	**S**	**H**	**H**	**H**	**S**	**H**	**H**	**R**	**S**
52	JTS27-2 × SN-20	**H**	**S**	**S**	**R**	**R**	**R**	**H**	**H**	**H**	**H**	**S**	**H**	**H**	**R**	**S**
53	JTS28-4 × SN-20	**S**	**S**	**H**	**R**	**R**	**S**	**H**	**H**	**H**	**H**	**S**	**H**	**H**	**R**	**S**
54	JTS33-3 × SN-20	**H**	**H**	**R**	**R**	**R**	**R**	**H**	**H**	**H**	**H**	**S**	**H**	**H**	**H**	**R**
55	JTS35-4 × SN-20	**H**	**H**	**H**	**R**	**R**	**R**	**H**	**H**	**H**	**H**	**S**	**H**	**H**	**H**	**R**
56	JTS37-4 × SN-20	**H**	**S**	**H**	**R**	**R**	**R**	**H**	**H**	**H**	**S**	**S**	**H**	**H**	**H**	**S**
57	JTS01-3 × SN-33	**S**	**S**	**S**	**R**	**S**	**R**	**H**	**H**	**H**	**H**	**S**	**H**	**H**	**R**	**S**
58	JTS05-2 × SN-33	**H**	**S**	**R**	**H**	**S**	**R**	**S**	**H**	**H**	**H**	**S**	**H**	**H**	**R**	**S**
59	JTS07-2 × SN-33	**H**	**S**	**S**	**R**	**S**	**R**	**H**	**H**	**H**	**H**	**S**	**H**	**R**	**H**	**S**
60	JTS09-4 × SN-33	**H**	**S**	**R**	**R**	**R**	**R**	**H**	**H**	**H**	**H**	**S**	**H**	**R**	**H**	**S**
61	JTS11-4 × SN-33	**H**	**S**	**S**	**R**	**R**	**R**	**H**	**H**	**H**	**H**	**S**	**H**	**H**	**H**	**S**
62	JTS35-3 × SN-33	**H**	**S**	**S**	**R**	**S**	**S**	**H**	**H**	**H**	**H**	**S**	**H**	**H**	**R**	**S**
63	JTS37-3 × SN-33	**H**	**S**	**R**	**R**	**R**	**R**	**H**	**H**	**H**	**H**	**S**	**H**	**H**	**H**	**S**
64	JTS21-3 × SN-33	**H**	**H**	**R**	**R**	**S**	**R**	**H**	**H**	**H**	**H**	**S**	**H**	**H**	**R**	**S**
65	JTS25-4 × SN-33	**H**	**H**	**S**	**R**	**R**	**R**	**H**	**H**	**H**	**H**	**S**	**H**	**H**	**R**	**S**
66	JTS25-5 × SN-33	**H**	**S**	**S**	**H**	**R**	**R**	**S**	**H**	**H**	**H**	**S**	**H**	**H**	**R**	**S**
67	JTS26-3 × SN-33	**H**	**S**	**S**	**R**	**R**	**R**	**H**	**H**	**H**	**H**	**S**	**H**	**H**	**R**	**S**
68	JTS27-2 × SN-33	**H**	**S**	**S**	**R**	**R**	**R**	**H**	**H**	**H**	**H**	**S**	**H**	**H**	**R**	**S**
69	JTS28-4 × SN-33	**S**	**S**	**R**	**R**	**R**	**S**	**H**	**H**	**H**	**H**	**S**	**H**	**H**	**R**	**S**
70	JTS33-3 × SN-33	**H**	**H**	**R**	**R**	**R**	**R**	**H**	**H**	**H**	**H**	**S**	**H**	**H**	**H**	**R**
71	JTS37-4 × SN-33	**H**	**H**	**R**	**R**	**R**	**R**	**H**	**H**	**H**	**H**	**S**	**H**	**H**	**H**	**R**
	Interspecific rootstock (*S. l* × *S. l*)															
72	JTS01-3 × JTS33-3	**H**	**H**	**S**	**R**	**H**	**R**	**R**	**R**	**S**	**R**	**S**	**S**	**S**	**H**	**H**
73	JTS05-2 × JTS33-3	**H**	**H**	**R**	**H**	**H**	**R**	**H**	**R**	**S**	**R**	**S**	**S**	**S**	**H**	**R**
74	JTS07-2 × JTS33-3	**H**	**H**	**H**	**R**	**H**	**R**	**R**	**R**	**S**	**R**	**S**	**S**	**S**	**S**	**H**
75	JTS09-4 × JTS33-3	**H**	**H**	**R**	**R**	**R**	**R**	**R**	**R**	**S**	**R**	**S**	**S**	**S**	**S**	**H**
76	JTS11-4 × JTS33-3	**H**	**H**	**H**	**R**	**R**	**R**	**R**	**R**	**S**	**R**	**S**	**S**	**S**	**S**	**H**
77	JTS16-3 × JTS33-3	**H**	**H**	**H**	**H**	**H**	**R**	**H**	**R**	**S**	**R**	**S**	**S**	**S**	**S**	**H**
78	JTS35-3 × JTS33-3	**H**	**H**	**H**	**R**	**H**	**H**	**R**	**R**	**S**	**R**	**S**	**S**	**S**	**H**	**H**
79	JTS37-3 × JTS33-3	**H**	**H**	**R**	**R**	**R**	**R**	**R**	**R**	**S**	**R**	**S**	**S**	**S**	**S**	**H**
80	JTS25-5 × JTS33-3	**H**	**R**	**H**	**H**	**R**	**R**	**H**	**R**	**S**	**R**	**S**	**S**	**S**	**H**	**H**
81	JTS28-4 × JTS33-3	**H**	**H**	**R**	**R**	**R**	**H**	**R**	**R**	**S**	**R**	**S**	**S**	**S**	**H**	**R**
82	JTS37-4 × JTS33-3	**H**	**H**	**R**	**R**	**R**	**R**	**R**	**R**	**S**	**H**	**S**	**S**	**S**	**S**	**H**
83	JTS01-3 × JTS35-4	**H**	**H**	**H**	**R**	**H**	**R**	**R**	**R**	**S**	**R**	**S**	**S**	**S**	**H**	**H**
84	JTS05-2 × JTS35-4	**H**	**H**	**R**	**H**	**H**	**R**	**H**	**R**	**S**	**R**	**S**	**S**	**S**	**H**	**R**
85	JTS07-2 × JTS35-4	**H**	**H**	**H**	**R**	**H**	**R**	**R**	**R**	**S**	**R**	**S**	**S**	**H**	**S**	**H**
86	JTS09-4 × JTS35-4	**H**	**H**	**R**	**R**	**R**	**R**	**R**	**R**	**S**	**R**	**S**	**S**	**H**	**S**	**H**
87	JTS11-4 × JTS35-4	**H**	**H**	**H**	**R**	**R**	**R**	**R**	**R**	**S**	**R**	**S**	**S**	**S**	**S**	**H**
88	JTS16-3 × JTS35-4	**H**	**H**	**H**	**H**	**H**	**R**	**H**	**R**	**S**	**R**	**S**	**S**	**S**	**S**	**H**
89	JTS35-3 × JTS35-4	**S**	**H**	**H**	**R**	**H**	**H**	**R**	**R**	**S**	**R**	**S**	**S**	**S**	**H**	**R**
90	JTS37-3 × JTS35-4	**H**	**H**	**R**	**R**	**R**	**R**	**R**	**R**	**S**	**R**	**S**	**S**	**S**	**S**	**H**
91	JTS21-3 x JTS35-4	**H**	**R**	**R**	**R**	**H**	**R**	**R**	**R**	**S**	**R**	**S**	**S**	**S**	**H**	**H**
92	JTS25-4 × JTS35-4	**H**	**R**	**H**	**R**	**R**	**R**	**R**	**R**	**S**	**R**	**S**	**S**	**S**	**H**	**H**
93	JTS25-5 × JTS35-4	**H**	**R**	**H**	**H**	**R**	**R**	**H**	**R**	**S**	**R**	**S**	**S**	**S**	**H**	**H**
94	JTS27-2 × JTS35-4	**H**	**H**	**H**	**R**	**R**	**R**	**R**	**R**	**S**	**R**	**S**	**S**	**S**	**H**	**H**
95	JTS28-4 × JTS35-4	**S**	**H**	**R**	**R**	**R**	**H**	**R**	**R**	**S**	**R**	**S**	**S**	**S**	**H**	**H**
96	JTS37-4 × JTS35-4	**H**	**H**	**R**	**R**	**R**	**R**	**R**	**R**	**S**	**H**	**S**	**S**	**S**	**S**	**H**
97	Maxifort (Control)	**R**	**S**	**H**	**R**	**S**	**R**	**S**	**S**	**H**	**H**	**S**	**H**	**R**	**S**	**R**

I2 and I3: *Fusarium wilt*, Ve2: *Verticillium wilt*, J3: *Fusarium crown* and root rot, py1; Corky root rot, Mi23: Root-Knot nematode, Bw6 and Bw12: *Bacterial wilt*, Sw5: TSWV (*Tomato Spotted wilt virus*), Tm2a: ToMV (*Tomato mosaic virus*), Ty1 and Ty2: TYLCV (*Tomato yellow leaf curl virus*), Ph3: Late blight, Sm-565: Gray leaf spot, Cf9: Leaf mold. R: resistant, S: susceptible, H: heterozygous, B: bacteria, F: fungus, V: virus, N: nematode.

**Table 4:** List of horticultural traits of hybrid new combinations and commercial rootstock (Maxifort).

**No**	**Name**	**Germination**		**30 (DAS)**		**60 (DAT)**									
				SSD	SL	Plant Stem Diameter		PH	Internode Length from the Base to the Leaf					TRL	RFM
				(mm)	(cm)	(mm)		(cm)	(cm)					(cm)	(g)
		GP (%)	GV	Cotyledon to 1st		Cotyledon to 1st	9th to 10th		3rd	5th	7th	9th	11th		
	Interspecific rootstock (*S. l* × *S. h*)														
1	JTS01-3 × SN-42	87	7	4.97	19	14.31	17.43	295	8	14	22	35	49	93	281.61
2	JTS05-2 × SN-42	88	7	4.56	20	13.98	16.66	298	12	19	28	41	61	89	279.86
3	JTS07-2 × SN-42	87	5	3.82	21	12.99	15.77	290	17	31	50	77	106	72	267.21
4	JTS09-4 × SN-42	97	5	3.91	14	13.75	16.51	275	14	24	38	56	84	85	285.25
5	JTS11-4 × SN-42	97	5	3.51	19	13.4	15.79	280	9	14	22	38	58	82	291.05
6	JTS16-3 × SN-42	87	5	4.38	18	14.48	17.78	270	11	27	42	66	88	92	271.68
7	JTS21-3 × SN-42	97	7	5.21	23	15.04	19.9	310	15	23	38	54	79	101	358.15
8	JTS25-4 × SN-42	90	7	4.08	21	13.41	16.22	294	15	18	29	46	66	99	395.87
9	JTS25-5 × SN-42	97	5	3.86	18	13.91	15.9	290	12	19	31	49	69	74	297.25
10	JTS27-2 × SN-42	77	3	3.91	16	14.53	15.14	255	12	25	43	64	87	88	312.89
11	JTS28-4 × SN-42	37	3	4.02	15	15.56	17.62	280	9	15	25	40	63	89	385.91
12	JTS35-4 × SN-42	77	3	3.87	19	15.56	17.07	285	10	18	30	49	74	89	312.58
13	JTS37-4 × SN-42	37	3	3.68	20	12.18	15.16	290	15	27	44	68	98	73	294.25
14	JTS01-3 × SN-06	93	5	3.98	23	13.36	15.54	295	13	22	38	56	80	97	412.32
15	JTS05-2 × SN-06	99	5	3.87	15	13.47	16.75	265	13	18	28	42	65	74	275.35
16	JTS09-4 × SN-06	99	5	3.72	16	13.73	16.51	260	15	23	39	57	80	91	301.24
17	JTS11-4 × SN-06	97	5	3.94	21	13.37	16.84	305	12	20	33	50	69	84	281.21
18	JTS16-3 × SN-06	93	5	4.16	20	15.08	17.33	295	10	18	30	48	69	85	287.21
19	JTS35-3 × SN-06	97	5	3.86	22	15.11	16.62	280	14	21	34	50	71	91	312.21
20	JTS37-3 × SN-06	80	5	4.34	17	14.03	16.48	270	11	18	33	52	74	73	259.98
21	JTS21-3 × SN-06	93	5	4.51	20	14.06	15.61	316	16	25	44	68	106	85	275.21
22	JTS25-5 × SN-06	93	5	4.35	18	15.12	15.15	270	10	21	35	53	77	91	285.35
23	JTS26-3 × SN-06	99	5	3.97	17	16.4	15.74	275	8	14	23	39	59	91	298.64
24	JTS28-4 × SN-06	70	3	4.08	18	14.6	16.71	280	10	17	28	42	66	97	321.55
25	JTS33-3 × SN-06	20	1	3.91	18	13.4	15.06	265	9	20	38	61	88	101	358.78
26	JTS35-4 × SN-06	60	1	3.89	20	12.4	16.15	260	12	22	40	62	88	85	375.25
27	JTS01-3 × SN-08	90	7	4.48	23	14.82	15.68	310	23	39	58	85	104	98	453.21
28	JTS05-2 × SN-08	93	5	4.37	19	16.19	17.13	250	10	16	28	43	70	78	310.54
29	JTS07-2 × SN-08	93	5	3.86	24	13.14	14.81	295	13	22	37	58	85	81	297.33
30	JTS09-4 × SN-08	80	5	3.59	17	14.95	15.01	275	9	15	25	40	61	103	324.22
31	JTS11-4 × SN-08	73	5	3.49	18	15.71	16.96	310	10	19	35	53	73	91	310.58
32	JTS16-3 × SN-08	60	3	3.75	15	13.69	15.17	265	11	16	26	40	61	78	298.31
33	JTS35-3 × SN-08	87	5	3.76	18	14.48	15.26	270	12	21	33	49	70	102	405.21
34	JTS37-3 × SN-08	87	5	3.68	19	14.01	17.51	250	10	18	30	50	80	94	412.21
35	JTS21-3 × SN-08	83	5	3.86	18	15.1	19.9	305	10	23	37	54	80	103	395.01
36	JTS25-4 × SN-08	97	5	3.75	18	14.9	17.13	275	6	9	15	23	36	69	261.23
37	JTS25-5 × SN-08	53	3	3.59	15	15.02	17.32	275	12	19	32	49	72	98	387.36
38	JTS26-3 × SN-08	43	1	3.21	17	13.02	15.29	245	9	18	30	47	66	86	297.53
39	JTS27-2 × SN-08	97	5	3.84	18	14.3	17.35	260	12	20	32	48	65	92	381.32
40	JTS28-4 × SN-08	88	7	4.12	13	14.5	18.01	270	8	14	19	30	45	110	476.38
41	JTS33-3 × SN-08	23	1	3.51	13	13.6	16.46	270	10	17	30	45	66	91	297.52
42	JTS35-4 × SN-08	23	1	3.29	15	14.8	15.82	280	9	15	28	47	79	79	291.9
43	JTS01-3 × SN-20	77	5	3.83	20	15.01	17.76	330	13	24	41	59	82	68	308.56
44	JTS05-2 × SN-20	87	5	3.51	17	15.3	17.06	240	10	16	28	42	62	89	298.55
45	JTS07-2 × SN-20	73	5	3.2	15	14.7	18.72	335	13	35	52	80	110	95	352.15
46	JTS11-4 × SN-20	97	5	4.01	19	14.01	15.83	310	10	20	33	49	69	72	311.59
47	JTS16-3 × SN-20	90	5	3.84	18	14.45	16.79	300	8	14	24	40	59	81	359.9
48	JTS37-3 × SN-20	80	3	3.52	17	14.3	15.72	310	9	15	29	51	80	78	372.16
49	JTS21-3 × SN-20	67	3	3.87	21	16.3	20.58	325	10	20	35	52	76	70	319.48
50	JTS25-4 × SN-20	70	3	3.24	16	14.01	16.2	290	8	13	21	35	50	73	325.45
51	JTS25-5 × SN-20	50	3	3.12	14	14.5	15.91	315	10	17	29	48	70	85	293.24
52	JTS27-2 × SN-20	57	3	3.54	16	13.7	15.79	300	8	13	21	37	59	98	298.11
53	JTS28-4 × SN-20	47	3	3.21	17	15.05	17.47	285	7	12	20	36	59	81	287.56
54	JTS33-3 × SN-20	73	5	3.29	19	15.3	15.71	290	12	20	32	52	77	105	345.61
55	JTS35-4 × SN-20	40	1	3.76	17	15	15.65	300	12	24	42	65	95	81	301.25
56	JTS37-4 × SN-20	60	3	3.25	15	14.3	15.47	285	11	18	29	44	70	92	342.68
57	JTS01-3 × SN-33	80	5	3.98	20	16.12	18.66	294	11	16	24	38	55	102	475.31
58	JTS05-2 × SN-33	90	3	3.45	18	14.95	15.48	215	13	22	35	53	72	112	489.25
59	JTS07-2 × SN-33	97	5	3.91	18	15.8	18.25	280	11	16	25	39	60	95	375.56
60	JTS09-4 × SN-33	90	5	3.35	15	17.76	18.78	245	11	17	29	45	66	85	365.23
61	JTS11-4 × SN-33	99	5	3.19	18	15.11	19	260	10	16	27	42	63	102	405.85
62	JTS35-3 × SN-33	99	5	3.18	19	15.7	19.21	240	13	17	24	35	48	89	425.31
63	JTS37-3 × SN-33	93	3	3.61	18	15.1	17.39	240	12	19	31	49	73	97	431.25
64	JTS21-3 × SN-33	99	7	4.25	22	15.35	20.7	270	12	18	25	36	50	92	348.53
65	JTS25-4 × SN-33	90	5	3.46	18	14.2	17.47	235	11	16	24	36	57	71	293.18
66	JTS25-5 × SN-33	83	3	4.03	12	15.6	20.69	230	11	16	24	38	56	96	274.4
67	JTS26-3 × SN-33	67	3	3.19	14	15.5	17.84	225	13	24	42	58	79	107	397.21
68	JTS27-2 × SN-33	98	5	4.01	16	17.5	19.83	225	9	15	25	39	57	98	305.98
69	JTS28-4 × SN-33	70	3	3.95	17	17.3	20.49	245	9	16	28	45	67	87	468.49
70	JTS33-3 × SN-33	77	5	3.91	15	16.7	20	245	10	15	24	37	57	90	435.52
71	JTS37-4 × SN-33	27	1	3.01	15	14.3	17.27	240	8	20	39	63	90	83	334.21
	Intraspecific rootstock (*S. l* × *S. l*)														
72	JTS01-3 × JTS33-3	100	9	5.24	19	14.29	17.92	190	10	20	27	35	48	59	144.67
73	JTS05-2 × JTS33-3	100	9	4.21	18	11.6	17.14	156	12	24	31	44	51	51	125.89
74	JTS07-2 × JTS33-3	100	9	4.02	22	10	15.83	200	20	29	37	47	63	61	165.17
75	JTS09-4 × JTS33-3	100	9	4.68	20	12.5	14.37	185	18	26	37	52	68	45	134.89
76	JTS11-4 × JTS33-3	100	9	4.56	21	9.2	14.93	200	22	27	36	48	64	49	150.28
77	JTS16-3 × JTS33-3	100	9	4.58	19	10.2	17.11	165	16	23	31	42	55	67	171.95
78	JTS35-3 × JTS33-3	98	7	5.12	24	13.5	19.9	185	15	21	30	39	56	68	189.21
79	JTS37-3 × JTS33-3	100	9	4.31	21	10.6	15.15	180	14	22	30	38	51	59	189.25
80	JTS25-5 × JTS33-3	100	7	3.98	17	12	14.8	120	16	22	30	39	50	47	123.89
81	JTS28-4 × JTS33-3	100	9	4.21	20	10.4	13.8	160	17	23	32	42	60	73	215.98
82	JTS37-4 × JTS33-3	100	9	4.35	22	10.8	17.6	170	15	24	33	44	59	45	128.96
83	JTS01-3 × JTS35-4	100	9	4.57	24	8.03	11.63	185	16	23	29	39	53	59	121.98
84	JTS05-2 × JTS35-4	100	9	4.67	21	13.07	16.93	150	15	22	30	38	50	63	141.39
85	JTS07-2 × JTS35-4	100	9	4.02	19	10.06	14.07	209	16	23	32	50	66	71	213.08
86	JTS09-4 × JTS35-4	100	9	3.96	17	11.05	15.93	182	16	24	31	41	51	47	128.98
87	JTS11-4 × JTS35-4	100	7	5.31	20	11.86	17.61	196	14	24	33	43	54	68	222.52
88	JTS16-3 × JTS35-4	98	9	4.68	20	8.78	14.69	167	15	20	27	39	54	63	208.61
89	JTS35-3 × JTS35-4	100	7	4.21	19	12.03	19.8	187	18	25	32	42	54	73	268.68
90	JTS37-3 × JTS35-4	100	7	4.29	17	12.01	16.88	177	11	18	21	31	43	69	198.31
91	JTS21-3 × JTS35-4	100	9	4.01	20	11.02	16.21	214	18	27	37	49	67	75	251.91
92	JTS25-4 × JTS35-4	100	9	4.08	18	11.03	17.6	192	19	26	35	44	55	42	153.29
93	JTS25-5 × JTS35-4	100	9	5.01	19	10.05	19.51	161	16	24	33	42	53	72	142.67
94	JTS27-2 ×JTS35-4	100	9	4.81	18	11	16.92	137	16	23	34	44	58	71	198.9
95	JTS28-4 × JTS35-4	98	9	4.25	17	11.06	15.98	186	13	22	30	42	60	63	208.37
96	JTS37-4 × JTS35-4	100	9	3.91	17	8.07	12.67	203	16	25	35	50	61	49	125.28
97	Maxifort (Control)	85	7	4.25	23	15.2	20.16	257	11	17	26	39	57	75	258.56

SL: seedling length, PH: plant height, SSD: seedling stem diameter, TRL: total root length, RFM: root fresh mass, DAS: days after sowing, DAT: days after transplanting, GP: germination percentage, GV: germination vigor (note 1 = very weak, 2 = very weak to weak 3 = weak, 4 = weak to medium, 5 = medium, 6 = medium to strong, 7 = strong, 8 = strong to very strong, 9 = very strong) [34].
